# Evaluation of somatic copy number variation detection by NGS technologies and bioinformatics tools on a hyper-diploid cancer genome

**DOI:** 10.1186/s13059-024-03294-8

**Published:** 2024-06-20

**Authors:** Daniall Masood, Luyao Ren, Cu Nguyen, Francesco G. Brundu, Lily Zheng, Yongmei Zhao, Erich Jaeger, Yong Li, Seong Won Cha, Aaron Halpern, Sean Truong, Michael Virata, Chunhua Yan, Qingrong Chen, Andy Pang, Reyes Alberto, Chunlin Xiao, Zhaowei Yang, Wanqiu Chen, Charles Wang, Frank Cross, Severine Catreux, Leming Shi, Julia A. Beaver, Wenming Xiao, Daoud M. Meerzaman

**Affiliations:** 1 Office of Oncologic Diseases, Office of New Drug, Center for Drug Evaluation and Research, Food and Drug Administration, 10903 New Hampshire Avenue, Silver Spring, 20993 USA; 2 State Key Laboratory of Genetic Engineering, Human Phenome Institute, School of Life Sciences and Shanghai Cancer Center, Fudan University, Shanghai, China; 3 Computational Genomics and Bioinformatics Branch, Center for Biomedical Informatics and Information Technology (CBIIT), National Cancer Institute, Rockville, MD USA; 4 Illumina Inc., San Diego, CA USA; 5 Sequencing Facility Bioinformatics Group, Biomedical Informatics and Data Science Directorate, Frederick National Laboratory for Cancer Research, Frederick, MD USA; 6 Bionano Genomics, San Diego, CA 20892 USA; 7 National Center for Biotechnology Information, National Librarssy of Medicine, National Institutes of Health, Bethesda, MD 20892 USA; 8 Center for Genomics, Loma Linda University School of Medicine, Loma Linda, CA USA; 9 Oncology Center of Excellence, Food and Drug Administration, Silver Spring, MD USA

**Keywords:** Copy number variation, Cancer genome, Next-generation sequencing, Bioinformatics tools, Consistency, Accuracy, Reproducibility, Detection sensitivity, Genome ploidy

## Abstract

**Background:**

Copy number variation (CNV) is a key genetic characteristic for cancer diagnostics and can be used as a biomarker for the selection of therapeutic treatments. Using data sets established in our previous study, we benchmark the performance of cancer CNV calling by six most recent and commonly used software tools on their detection accuracy, sensitivity, and reproducibility. In comparison to other orthogonal methods, such as microarray and Bionano, we also explore the consistency of CNV calling across different technologies on a challenging genome.

**Results:**

While consistent results are observed for copy gain, loss, and loss of heterozygosity (LOH) calls across sequencing centers, CNV callers, and different technologies, variation of CNV calls are mostly affected by the determination of genome ploidy. Using consensus results from six CNV callers and confirmation from three orthogonal methods, we establish a high confident CNV call set for the reference cancer cell line (HCC1395).

**Conclusions:**

NGS technologies and current bioinformatics tools can offer reliable results for detection of copy gain, loss, and LOH. However, when working with a hyper-diploid genome, some software tools can call excessive copy gain or loss due to inaccurate assessment of genome ploidy. With performance matrices on various experimental conditions, this study raises awareness within the cancer research community for the selection of sequencing platforms, sample preparation, sequencing coverage, and the choice of CNV detection tools.

**Supplementary Information:**

The online version contains supplementary material available at 10.1186/s13059-024-03294-8.

## Background

The advances of next generation sequencing (NGS) technologies over the past several years have led to numerous discoveries in human diseases, highlighting the role of genetic variation [1–5]. Somatic copy number variants (CNVs) represent variations in the copy numbers of a DNA sequence during cancer development, differing from an individual’s germline DNA. They play a crucial role in the initiation, progression, and metastasis of tumors [6, 7].

Many tools have been developed to call CNVs from NGS data. Given the rapid evolution of these CNV callers, it is essential to assess their performance against large and diverse datasets. This not only allows for an unbiased evaluation but also contributes to a better understanding of their strengths and weaknesses.

Published CNV callers appeared to be reliable in large-scale data where they were benchmarked. However, these tests were run only by the authors of each caller, and were not usually run on multiple datasets [3]. In another study, validated CNVs used were artificially generated, where input positions were modified to create CNVs [4].

Other benchmarks have been performed on small datasets with whole-exome sequencing (WES) on diploid genomes or germline CNVs only [2]. Another benchmarking study on germline CNVs used many different detection tools that had different calling methodologies [8]. This benchmarking study was done to compare WES and WGS data sets, with CNVs called from an array-based method used as a reference [8]. In the study, the authors recognized that the array data cannot be used as a final validating set of CNVs and tools using same CNV calling strategy usually had higher concordance. In general, the precision for CNV calling seemed poor, especially within WES sample sets even when the tool was specified for WES use [8]. Hence, the authors proposed that to get higher precision in CNV calling within multiple CNV calling tools, WGS data sets should be used for CNV detection [8].

Using comprehensive datasets established in our previous study on HCC1395, whose genome ploidy was determined as 2.85 [9–12], we established CNV high-confident calls and assessed how non-analytical and analytical factors affect the reproducibility, accuracy, and sensitivity of CNV detection. These datasets consist of data from whole-exome sequencing (WES) and whole-genome sequencing (WGS), as well as orthogonal methods including microarray and Bionano, thus allowing us to explore consistency of CNV calling across different technologies on a challenging genome. With comprehensive data sets capturing non-analytical and analytical factors, we also explored how factors, such as amount of input DNA, type of input sample (fresh and formalin-fixed paraffin-embedded (FFPE)), tumor purity, and CNV callers (ascatNgs [13], CNVkit [14], FACETS [15], DRAGEN [16], HATCHet [17], Control-FREEC [18, 19]), affected the calling of different CNV types: gain calls, loss calls, and LOH.

## Results

### Study design

**Fig. 1:**
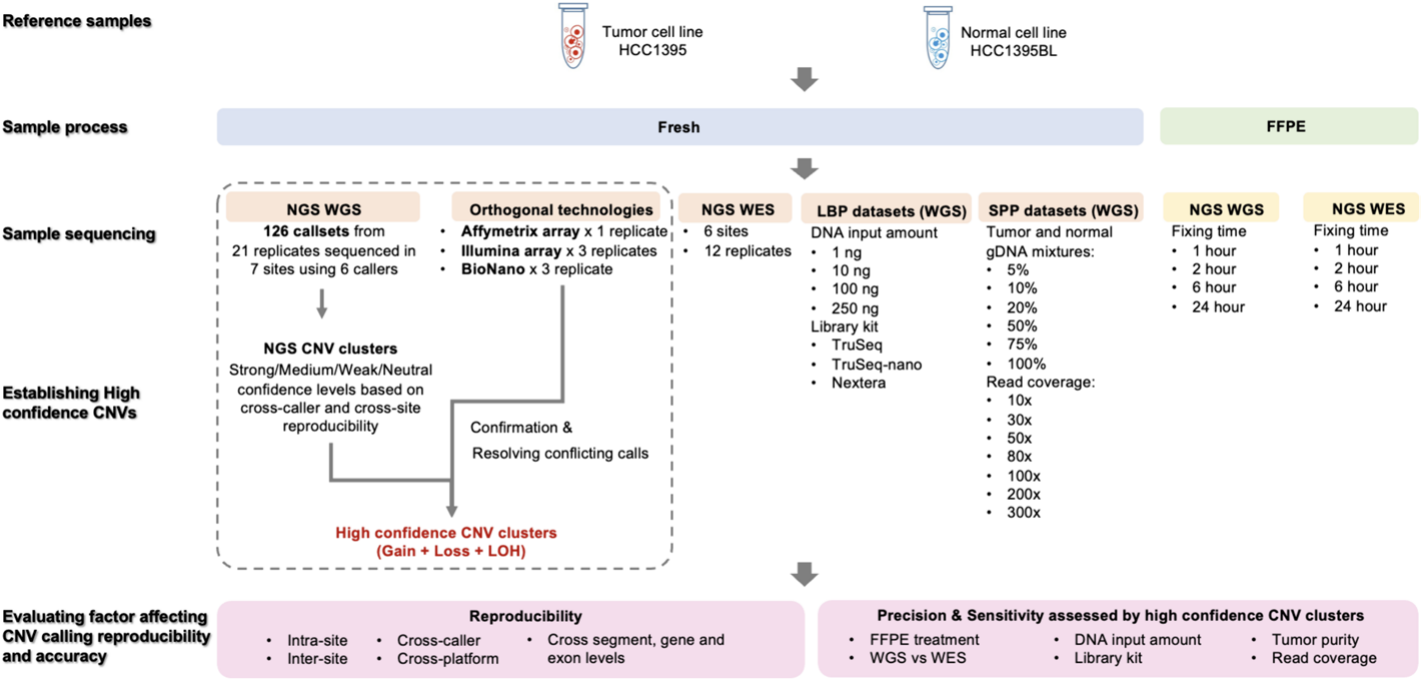
Overall study design. A total of 21 WGS replicates and 12 WES replicates performed across six sequencing centers was used to determine the consistency and reproducibility of CNV calling across sequencing centers, CNV callers, and sequencing platform (WGS vs WES). High-confidence CNV clusters in the cancer cell line were defined by the consensus scores across 21 WGS replicates with six callers (126), in combination of supporting evidence from three orthogonal technologies, Affymetrix CytoScan, Illumina Array, and Bionano. Finally, defined high-confidence CNV clusters were used to evaluate precision and detection sensitivity under various experimental conditions, such as amount of DNA input (1–250 ng), library preparation protocol (TruSeq, TruSeq-Nano, and Nextera Flex), tumor content range (5–100%), read coverage (10X–300X), and FFPE samples

This study rigorously evaluated six CNV callers: ascatNgs, CNVkit, FACETS, DRAGEN, HATCHet, and Control-FREEC. These callers underwent thorough evaluation in peer-reviewed studies [13–19] or were utilized in substantial cohort studies [20–22]. To comprehensively assess their performance, all callers underwent benchmarking using six NGS short-read datasets from the Somatic Mutation Working Group. These datasets were prepared under various experimental conditions, such as variations in input DNA amount, Fresh vs. FFPE samples, tumor purity, and the choice between WGS and WES [9] (Fig. 1).

Initially, to evaluate reproducibility across sequencing centers, callers, and platforms, the six CNV callers were applied to 21 WGS replicates sequenced across six different sequencing centers (Novartis (NV), Illumina-HiSeq (IL), Illumina-NovaSeq (NS), Fudan University (FD), European Infrastructure for Translational Medicine (EA), National Cancer Institute (NC), Loma Linda University (LL)). This resulted in 126 call sets (Fig. 1), stratified by center and caller, to assess concordance of CNV calls.

Subsequently, the callers were applied to a WES dataset comprising 12 replicates across six centers. A comparison between CNV calls from WGS and WES datasets was conducted to evaluate concordance across these different technologies. Using the WGS datasets, a reference CNV call set was established and validated against CNV calls derived from three orthogonal technologies: Affymetrix CytoScan, Illumina BeadChip, and Bionano.

Furthermore, to evaluate the impact of non-analytical factors, the six CNV callers were employed across following three datasets:

1. The WGS on library preparation protocol (LBP) dataset, encompassing samples prepared with three library preparation protocols (TruSeq, TruSeq-nano, Nextera flex) with varying DNA input amounts (ranging from 1 to 250 ng). This dataset assessed how DNA input amounts affected CNV calling.
2. The WGS on tumor sample purity (SPP) dataset, comprising varying tumor and normal gDNA mixtures with tumor purities ranging from 5 to 100% and different read coverage amounts (ranging from 10 to 300X coverage). This dataset explored the roles of tumor purity and read coverage in CNV calling.
3. The FFPE samples dataset, which included four time points for fixing time in both WGS and WES analyses. This dataset investigated how cell fixing time influenced CNV calling across the different callers.

### Concordance of CNV calls across six callers

**Fig. 2:**
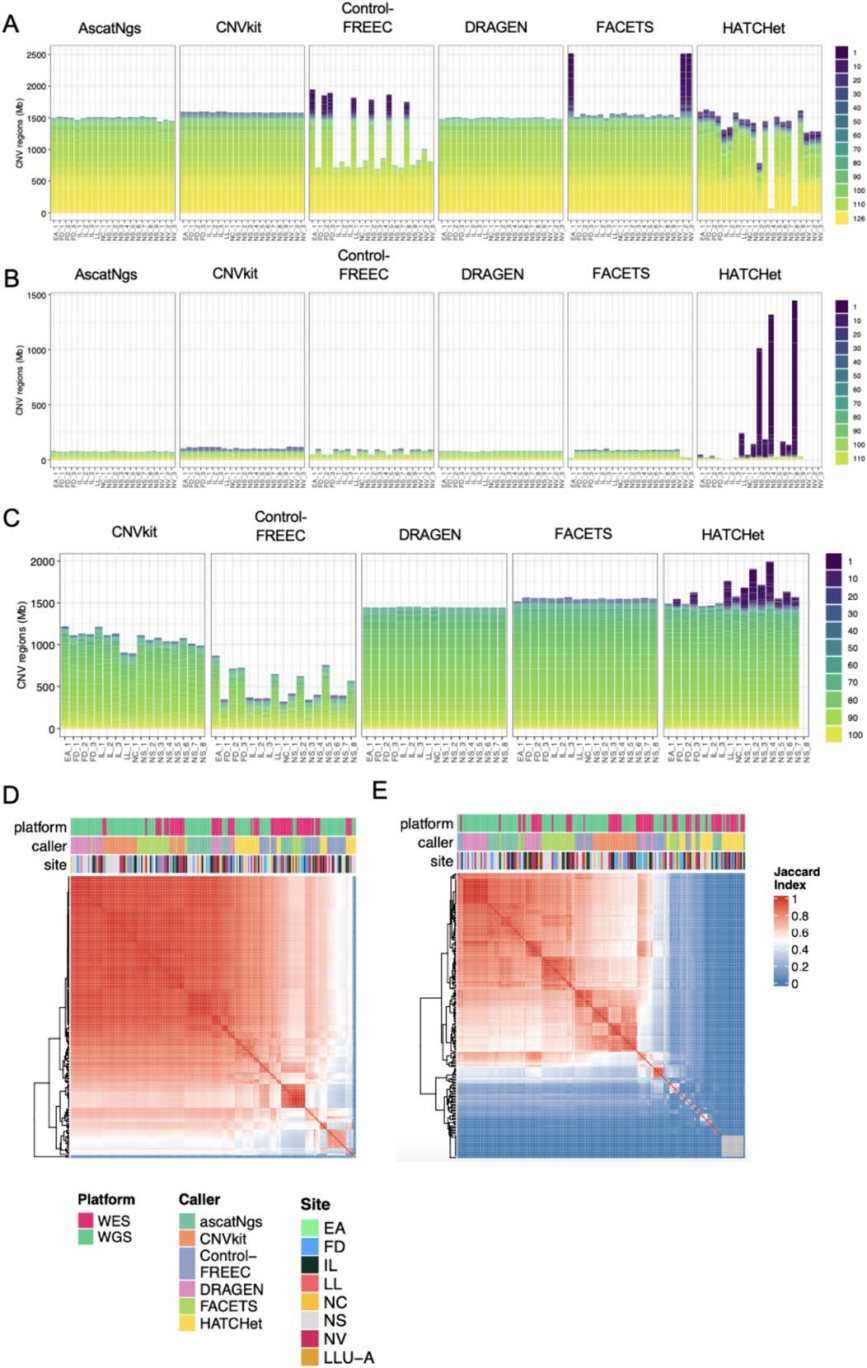
Consistency of CNV calls. **A** CNV gain regions, **B** CNV loss regions, and **C** CNV LOH regions supported by each replicate across five callers. Note, ascatNgs did not report LOH results. Heatmaps of Jaccard index of genome regions with gain (**D**) or loss (**E**) calls in 21 WGS and 12 WES replicates across six callers

Our evaluation focused on the consistency and reproducibility of CNV calls within WGS datasets generated by six callers, comprising 21 replicates from six sequencing centers. We initiated the examination by assessing the total genomic regions spanned by gain, loss, and LOH segments across replicates for each caller. ascatNgs, DRAGEN, and CNVkit consistently identified copy gains averaging about 1500 Mb (Additional file 1: Fig. S1A). These three callers maintained relative consistency in the copy number loss regions (Additional file 1: Fig. S1B). FACETS displayed reasonable consistency in gain and loss genome regions, except for a few outliers (Fig. 2A, Additional file 1: S1A). Conversely, HATCHet and Control-FREEC showed notable inconsistency across replicates in both gains and losses (Fig. 2A, Additional file 1: S1A). DRAGEN, Control-FREEC, and CNVkit reported fewer LOH regions compared to FACETS and HATCHet (Additional file 1: Fig. S1C). While CNVkit and DRAGEN maintained consistency around 1000–1500 Mb, the majority of FACETS and HATCHet calls surpassed the 1500-Mb range, with some larger outliers.

Further examination of CNV regions across replicates and callers revealed that ascatNgs, CNVkit, and DRAGEN consistently exhibited the highest consensus in identifying CNV gains (Fig. 2A). In contrast, the remaining three callers produced a higher number of unique calls per replicate, particularly HATCHet, presenting the most unique gain regions. Similar trends were evident in the identified loss regions (Fig. 2B). DRAGEN and FACETS demonstrated higher consistency in LOH calls, with most CNV regions supported across 105 replicates (21 replicates × 5 callers) (Fig. 2C). Conversely, HATCHet showed inconsistencies across replicates, with numerous clusters specific to single or subsets of replicates (Fig. 2C). Comparing CNV calling results between WGS and WES through Jaccard indexes revealed that clustering was primarily influenced by the caller, followed by the platform (WGS vs WES), with minimal sequencing center impact (Fig. 2D, E). Stratifying comparisons by platform showed similar distributions of Jaccard indexes within and across sequencing centers, confirming minimal sequencing center impact (Additional file 1: Fig. S1D, E). Within WGS replicates, ascatNgs, CNVkit, and DRAGEN displayed the most consistency for gains and losses (Additional file 1: Fig. S1D, E). FACETS exhibited high concordance for most within WGS comparisons but lower concordance for some. Control-FREEC and HATCHet displayed the most variability.

Within WES replicates, all callers showed lower concordance compared to WGS, especially for losses (Additional file 1: Fig. S1D, E). CNVkit and DRAGEN maintained the highest concordance within WES replicates, while Control-FREEC and HATCHet demonstrated the lowest concordance for losses. Cross-platform comparisons revealed moderate concordance for gains and more variable, lower concordance for losses across all callers (Additional file 1: Fig. S1D, E). CNVkit and DRAGEN displayed the highest concordance for both gains and losses between WGS and WES.

Additionally, we measured concordance at segment, gene, and exon levels using the Jaccard Index for CNV gain and loss detected from WGS replicates. The intra-caller concordance surpassed inter-caller concordance overall. Notably, the heatmap for loss calls highlighted HATCHet as distinct from other callers, likely due to generating more unique loss calls. The distribution patterns within and across sequencing centers were akin, suggesting minimal impact on CNV calling when adhering to the same WGS library preparation protocol (Additional file 1: Fig. S2F, G).

### Inconsistency between replicates due to inaccurate assessment of genome ploidy

We then set to understand inconsistent CNV calling observed in the 21 WGS datasets by FACETS and HATCHet. These inconsistencies were likely due to an inaccurate assessment of genome ploidy by these callers, resulting in excessive gain or loss calls (Additional file 1: Fig. S3). Genome ploidy significantly influences how CNV callers identify gains or losses. Some callers assume the genome median coverage as copy-neutral (CN = 2), identifying segments with higher coverage than the median as gains and those with lower coverage as losses. However, this assumption may fail when the genome median coverage deviates significantly from the copy-neutral level, particularly in samples with numerous alterations.

The cell line used in this study, with an overall genome ploidy reported as 2.85 in literature [23], reflects the challenge of deviations from the typical diploid level observed in somatic samples [24]. CNVkit, one of the callers, was adjusted with manual re-centering based on this known genome ploidy. Manual re-centering becomes necessary when the overall genome ploidy differs significantly from the assumed diploid level (CN = 2) by CNVkit. Without this adjustment, CNVkit may call gains or losses using the median coverage, which could be inaccurate when the median coverage is far from the copy-neutral coverage. An instance was noted on chr21 where CNVkit, without manual re-centering, incorrectly identified a deletion (DEL) in a region with a B-Allele Frequency close to 50%, indicating compatibility with multiples of CN = 2 and no LOH. However, the genomic region had lower coverage than the median (approximating the overall genome ploidy, 2.85), leading CNVkit, without manual re-centering, to interpret it as a loss.

We also computed an examination of ploidy based on Control-FREEC calling results (see “Methods”). In the seven WGS runs with excessive gain calls (as depicted in Fig. 2A), they consistently revealed a ploidy level of 5 (as shown in Additional file 1: Table S1). These discrepancies underscore the critical importance of comprehending and adjusting for genome ploidy variations, particularly within CNV calling methodologies, to prevent misinterpretations when departing from the expected diploid level.

### Establishment of CNV reference call set for the *cancer* cell line (HCC1395)

From the analysis of the six CNV callers applied to the 21 WGS replicates dataset, we devised an integration strategy to construct a consensus CNV call set (Additional file 1: Fig. S4A). The process involved segmenting the data based on overlapping call sets to identify distinct segments (Additional file 1: Fig. S4B).

We established a scoring system to assess the confidence level of each CNV interval by evaluating the consistency of CNV calls across replicates and among different callers. The initial scoring involved assessing reproducibility across the 21 replicates for each of the six callers (Additional file 1: Fig. S5A). This scoring system ranged from 0 to 3, with higher scores indicating stronger consistency within the same callers. The total of these scores from all callers determined the confidence level of CNV intervals, categorized as strong, medium, weak, or neutral (Additional file 1: Fig. S5B). Higher confidence levels indicated stronger support from multiple sequencing centers and callers, reinforcing the reliability of the identified CNV intervals.

**Fig. 3:**
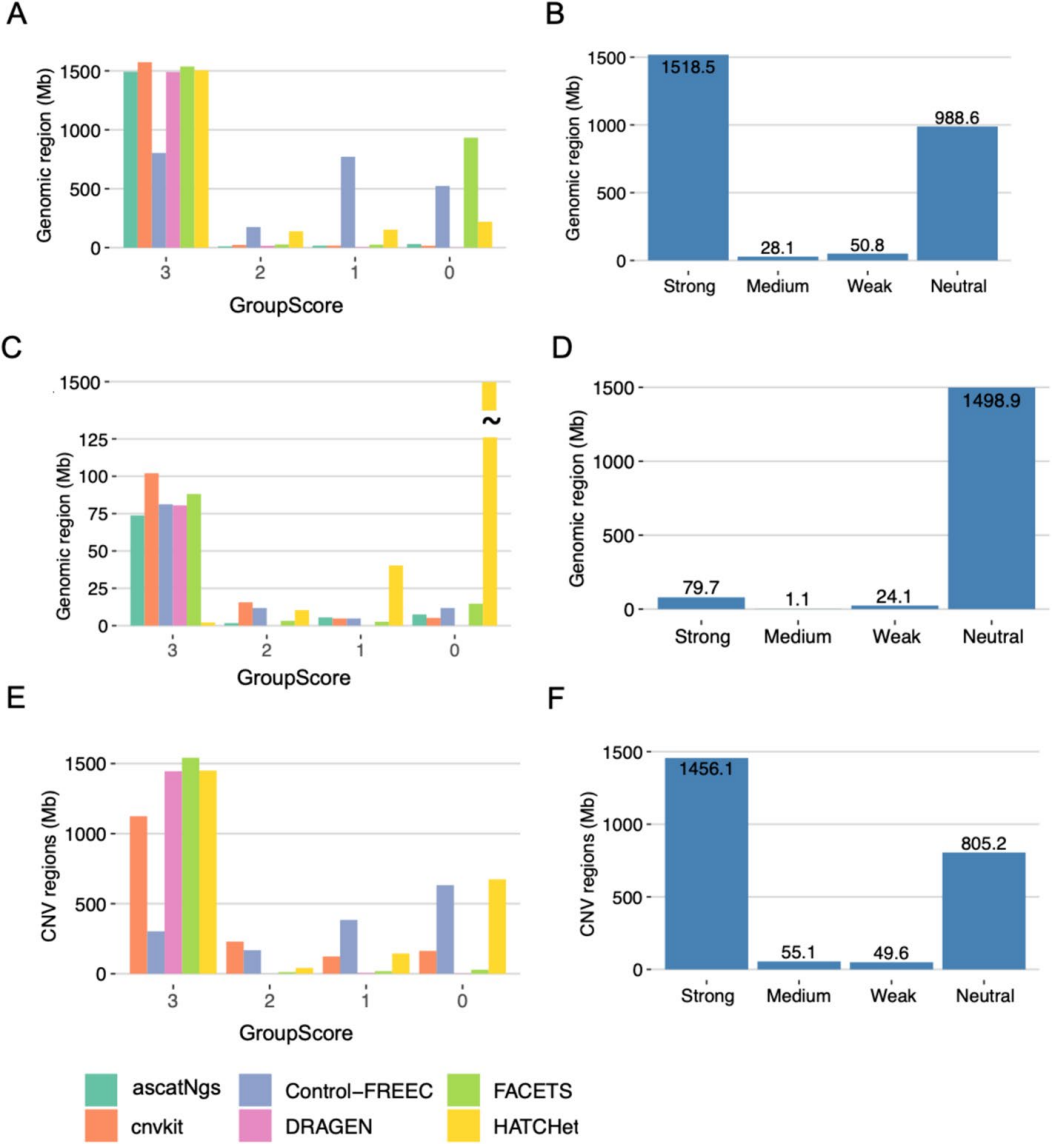
Genomic regions of CNV with site scores and confidence levels. Genomic region of copy number gain called by each caller with site scores (**A**) and confidence levels (**B**), copy number loss (**C** and **D**), and LOH (**E** and **F**)

Upon examination, most CNV calls for gains, losses, and LOH intervals received a group score of 3, signifying reproducibility within and across groups using the same caller. However, inconsistencies were observed for specific calls between replicates: Control-FREEC and FACETS for CNV gains (Fig. 3A), HATCHet for CNV losses (Fig. 3C), and Control-FREEC and HATCHet for LOH calls (Fig. 3E). Consequently, more CNV regions received lower scores (0 and 1) in these instances.

Further refinement utilized consensus evidence across all six callers to categorize CNV intervals into four confidence levels. Strong-evidence CNV intervals were widely supported by most replicates and callers, while weaker confidence levels had support from fewer replicates or specific callers. Neutral-evidence CNV intervals were only substantiated by specific sequencing centers or CNV callers. The resulting analysis showcased 1518.5 Mb of genome regions strongly supported for copy gains (Fig. 3B) and 1456.1 Mb for LOH regions (Fig. 3F). However, for loss intervals, the consensus evidence shifted away from strong support, with more regions associated with neutral evidence (1498.9 Mb), primarily backed by unique calls from HATCHet (Fig. 3D).

### Validation of CNV call sets with orthogonal methods

The consensus calls from the 126 WGS datasets were validated against CNV calls derived from three different orthogonal methods: Affymetrix CytoScan, Illumina BeadChip, and Bionano. This validation process involved comparing CNVs identified by these orthogonal methods with those in the consensus call set, categorized by confidence levels for both gain and loss clusters (Additional file 1: Fig. S4C).

**Fig. 4:**
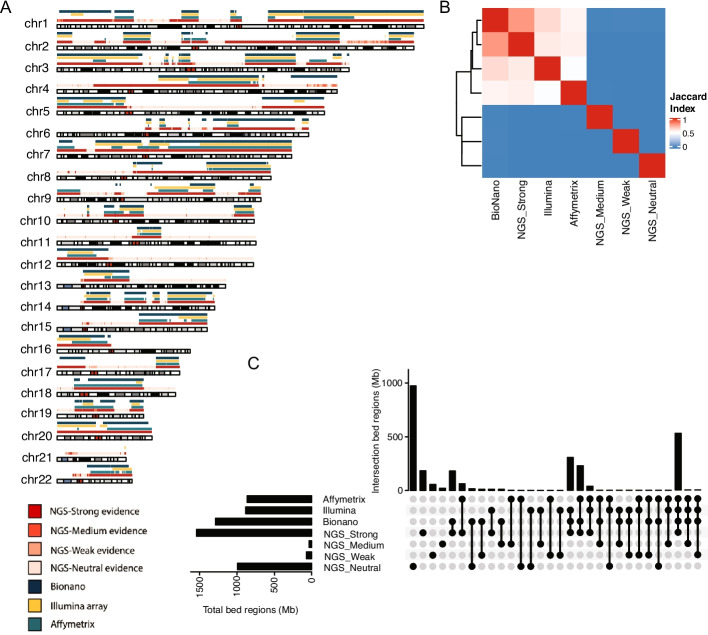
Consistency of collapsed gain clusters in comparison to three orthogonal technologies. **A** Chromosome view of gain intervals in comparison to three orthogonal technologies. **B** Paired-wise concordance of Jaccard index for gain intervals with different confidence level in comparison to three orthogonal technologies. **C** Upset plot of concordance of gain intervals with different confidence level in comparison to three orthogonal technologies. Intersections of different NGS confidence levels were not included

Strong-evidence gain calls exhibited a high validation rate of 88% when supported by at least one of the three orthogonal technologies (Table 1). Similarly, all strong- evidence loss calls were validated by at least one orthogonal technology. However, as the confidence level decreased, the validation rate of calls decreased accordingly. For CNV gains and LOH, the NGS strong-evidence regions were well supported by all three orthogonal technologies (Fig. 4 and Additional file 1: Fig. S7). Regarding CNV losses, Illumina BeadChip and Bionano corroborated the NGS strong-evidence regions, while Affymetrix CytoScan aligned more with NGS neutral-evidence regions (Additional file 1: Fig. S8). Nevertheless, the evidence from Affymetrix CytoScan alone was not substantial enough to solely support these CNV calls.

**Table 1:** Confirmation of CNV benchmark sets by three orthogonal methods (based on regions, Mb)

Confidence level	Collapsed calls from NGS	Affymetrix Cytoscan	Illumina array	Bionano	Validated by at least one technology	Validated by at least two technologies
Copy number gain						
Strong	1542.1	858.2	883.3	1243.8	1357.2 (88.0%)	1099.5 (72.3%)
Medium	35.0	3.1	0.3	11.7	14.6 (41.8%)	0.5 (1.3%)
Weak	69.1	0.1	0.1	12.9	13.0 (18.8%)	0.1 (0.1%)
Neutral	992.3	0.7	0.3	17.3	18.1 (1.8%)	0.2 (0.0%)
Copy number loss						
Strong	79.7	79.7	79.5	78.9	79.7 (100%)	79.6 (99.9%)
Medium	1.1	1.1	0.5	0.3	1.1 (94.9%)	0.5 (44.1%)
Weak	24.2	23.3	7.6	7.7	23.3 (96.1%)	7.7 (31.8%)
Neutral	1518.9	799.8	23.5	24.4	800.7 (52.7%)	24.5 (1.6%)

The CNV benchmark sets comprised strong-evidence calls and medium/weak- evidence calls supported by at least two of the three orthogonal technologies. Neutral-evidence calls were excluded from the benchmark set. Conflicting CNV regions, where gain and loss were identified simultaneously, were carefully handled: if a strong-evidence NGS CNV gain region was identified as a loss by orthogonal technologies and not supported as a gain by any, it was removed from the benchmark set. However, if gain calls were detected by orthogonal technologies in a strong-evidence NGS CNV gain region, even though other orthogonal technologies identified it as a loss, the region was retained in the benchmark set.

Furthermore, breakpoints were trimmed based on evidence from orthogonal technologies. If a gain end and a loss end overlapped and the gain end was supported by any orthogonal technologies, the gain end was retained, while the loss end was trimmed.

This rigorous validation process resulted in CNV benchmark sets for HCC1395, comprising 346 high-confidence gain calls spanning 1525.6 Mb, 33 high-confidence loss calls covering 87.9 Mb, and 320 high-confidence LOH calls encompassing 1490.4 Mb (Additional file 1: Table S2, Fig. S9).

### Effect of tumor content/FFPE/DNA inputs/library prep protocol on calling results

**Fig. 5:**
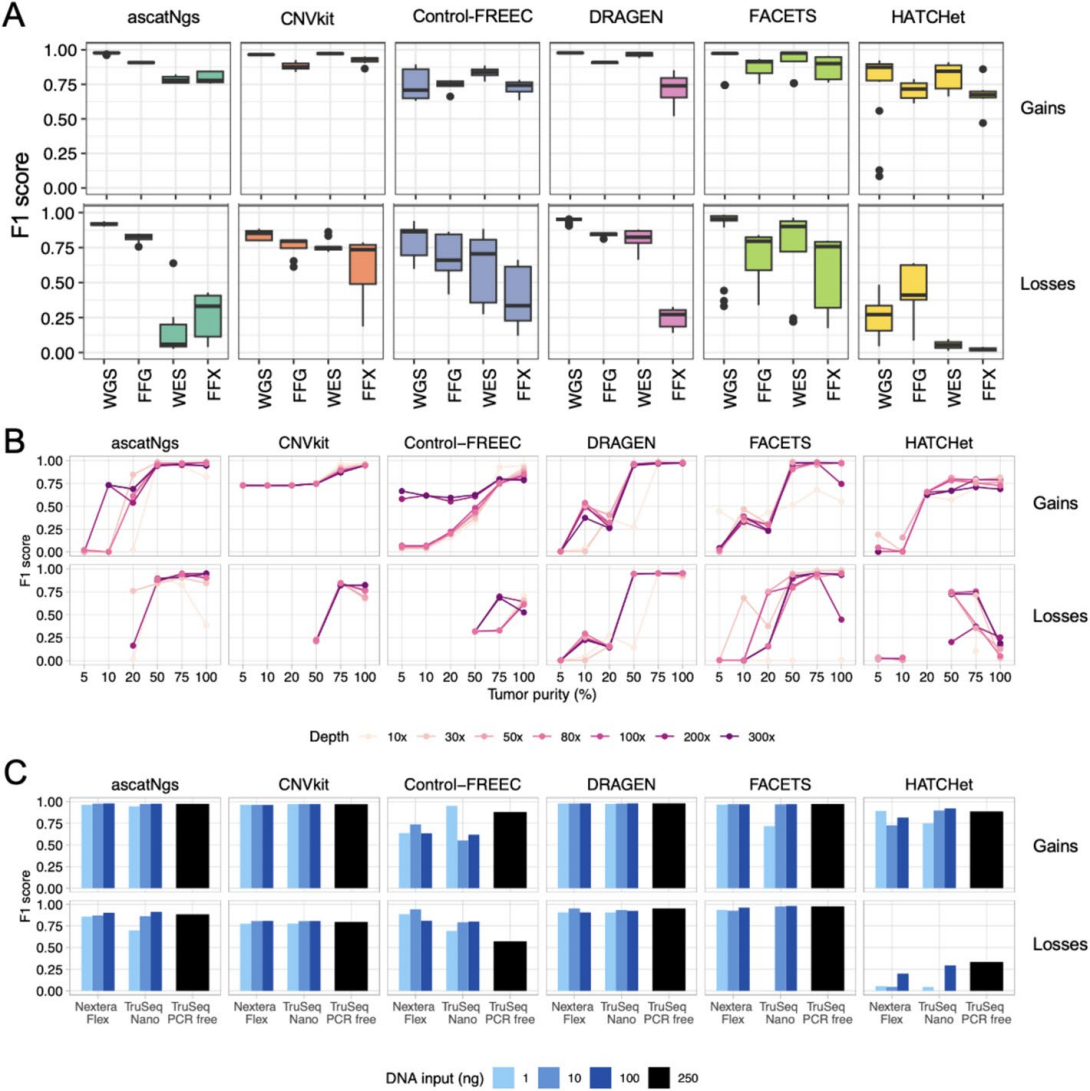
The accuracy of copy number gains and loss per genome regions from Fresh/FFPE, WGS/WES, tumor purity, and library preparation dataset. F1 score of gain and loss calls by six callers on Fresh/FFPE and WGS/WES (**A**), tumor purity dataset (**B**), and library preparation dataset (**C**). FFG: FFPE on WGS, FFX: FFPE on WES

To evaluate the influence of non-analytical factors on the performance of each CNV caller, we considered three datasets: FFPE, tumor content, DNA inputs, and library preparation. First, to assess the impact of FFPE, we applied all callers to the FFPE dataset that included four time points for fixing time (1, 2, 6, and 24 h), each sequenced with both WGS and WES. CNV results from WGS and WES of the FFPE samples (FFG and FFX, respectively) along with CNV results from WGS and WES of fresh samples were evaluated by calculating precision and recall, using the high-confidence call set as the truth. The accuracy of callers varied across both WGS/WES and FFPE/Fresh datasets, shown by the precision and recall of the six callers on fresh or FFPE DNA in WGS/WES replicates (Fig. 5A, Additional file 1: Fig. S10). Overall, we observed the impact of DNA damage due to FFPE process on the precision and recall of CNV calling by all six callers (Additional file 1: Fig. S10). In WGS data set, the precision of loss calls dropped significantly (*p*-value of 1.49e − 07, one-tailed *t*-test, Additional file 1: Fig. S10A). Even though CNV calling by ascatNgs, CNVkit, and DRAGEN were also impacted in FFPE data sets, their calling reproducibility remained consistent across replicates, particularly for WGS platform (Additional file 1: Fig. S10B-C). It is evident from the analysis that the increase in FFPE fixing time does not significantly impact the accuracy of CNV calls (Additional file 1: Fig. S11). Furthermore, the accuracy of CNV calls derived from FFPE samples is higher in WGS datasets compared to WES datasets. The accuracy is primarily dependent on the specific caller employed. CNVkit demonstrated relatively higher performance in detecting copy number gain, whereas HATCHet and Control-FREEC exhibited comparatively lower performance. DRAGEN achieved high performance in detecting both copy number gain and copy number loss, except for FFPE WES datasets.

To assess the effect of different tumor purity and read coverages on CNV calling, we analyzed the SPP dataset, which included samples with different combinations of tumor purity (5%, 10%, 20%, 50%, 75%, 100%) and read coverage (10X, 30X, 50X, 80X, 100X, 200X, 300X) (Fig. 5B). For ascatNgs and DRAGEN, calling accuracy of both gain and loss dropped drastically with tumor purity equal to or less than 20% and performance was not significant impacted by increasing read coverage if it was great than 30X. AscatNgs, CNVkit, Control-FREEC, and HATCHet failed in making any loss calls at low tumor content (< 20%).

To assess the impact of DNA input amount and library preparation methods, we used the LBP dataset, which consisted of samples with different combinations of DNA input (1, 10, 100, and 250 ng) and library kit (TruSeq, TruSeq-nano, Nextera). There were no significant differences between the different DNA input amounts and library preparation methods for results from ascatNgs, CNVkit, and DRAGEN (Fig. 5C). All callers were consistent in performance based on the F1 scores. Even low DNA input amounts achieved high performance in the copy number gain and loss calls. Inconsistent results were observed for Control-FREEC and HATCHet. FACETS achieved consistent results with different inputs with Nextera and 10 and 100 ng input with TruSeq-nano but did not produce any loss calls for data derived from 1 ng with TruSeq-nano protocol (Fig. 5C).

## Discussion

This study was performed on a comprehensive set of data, allowing for a detailed assessment of recent CNV detection tools. We analyzed the performance and reproducibility of the six different CNV callers across different factors such as sequencing center, WES vs WGS, tumor purity and read coverage, DNA input amount, and FFPE vs Fresh samples. Observed differences in results were not only based on the varying technologies but also the CNV callers.

Across different sequencing centers, using manufacturer-recommended protocols for library preparation and sequencing, notable variations in CNV calling were not observed. However, a significant impact was seen between WGS and WES platforms, with WGS being more accurate and reproducible compared to WES. It is important to note that certain CNV callers, like ascatNgs, might not be suitable for WES data due to platform differences. Additionally, the choice of CNV caller significantly influences accuracy, especially in assessing ploidy in cancer genomes.

It is worth noting that the impact of minor variations, like different random seed numbers used by ascatNgs, might lead to segment splitting in one run but will not significantly affect reference CNV call set definition in datasets with over 50X coverage. However, this variation might pose concerns when ascatNgs alone is used on datasets with 50X or lower coverage (Additional file 1: Fig. S12). Inverse correlation was observed between the variability of ascatNgs calling results and sequencing depth (Table S3).

Assessment of CNV calls from FFPE vs. fresh samples, DNA input amount, read coverage, and tumor purity highlighted decreased precision and recall of CNV calls by each caller across both sequencing platforms. This indicates a substantial impact of the FFPE process on CNV calling results in both WGS and WES datasets (Fig. 5A, Additional file 1: Fig. S10).

Interestingly, varying DNA input amounts did not notably affect CNV caller performance in copy number gain regions except for FACETS, which failed to make loss calls with data derived from TruSeq-Nano and 1 ng DNA input due to high read redundancy [23].

It worth to note that we previously defined structural variant (SV) call set encompassing various types, such as DEL (deletion), DUP (duplication), INS (insertion), INV (inversion), TRA (translocation), and BND (Complex Break Point Events) [12]. The SV detection callers primarily sought supporting reads for these events, focusing on higher resolutions that are more sensitive to sub-populations of cells. Previous studies have indicated the highly heterogeneous nature of the cancer cell line HCC1395, characterized by large subclones. Consequently, SV events occurring in smaller cell populations were incorporated into the final SV reference call set, resulting in a larger number of events defined in SV (1788) compared to CNV (688) (Additional file 2). Notably, the average sizes of DEL (deletion) and DUP (duplication), equivalent to LOSS and GAIN respectively, were considerably smaller [12]. In contrast, CNV callers primarily targeted the LOSS or GAIN of chromosome regions within overall cell populations, factoring in genome ploidy. Therefore, CNV calling exhibited lower sensitivity towards events represented by a small fraction of cells or a limited number of supporting reads. Consequently, we assert that the SV and CNV call sets should remain separate entities.

We also wish to point out that even though we provided copy number calls in the reference call set (Additional file 3, 4 and 5), our confidence was only limited to qualification of CNV calls, i.e., GAIN, LOSS, and LOH. The copy number calls which were derived from the median of called values by six callers should not be used as “gold-standard” for benchmarking study. In addition, break points of CNV segment were the best estimate and thus imprecise.

## Conclusions

This study benchmarked somatic CNV calling across real sequencing data, and different non-analytical factors and investigated how these different factors affected CNV calling. A high confident CNV call set, including copy gain, loss, and loss of heterozygosity, was established for the reference sample (Additional file 1: Fig. S9).

NGS technologies and current bioinformatics tools can offer reliable results for detection of copy gain, loss, and LOH. However, when working with a hyper-diploid genome, some software can call excessive copy gain or loss due to inaccurate assessment of genome ploidy. With performance matrices on various experimental conditions, this study raises awareness within the cancer research community for the selection of sequencing platforms, sample preparation, sequencing coverage, and the choice of CNV detection tools.

## Methods

### Implementation of somatic CNV detection algorithms

#### Ploidy-awareness for benchmarked methodologies

**Table:** 

	Purity/Ploidy	Version
ascatNgs	✓	4.2.1
CNVkit	Manual	0.9.1
Control-FREEC	✓	11.6
DRAGEN	✓	4.0.x
FACETS	✓	0.6.0
HATCHet	✓	1.0.4

#### CNVkit

Samples (WGS, WES, SPP, LBP, FFX, FFG) of SEQC-II triple-negative breast cancer cell line HCC1395 were analyzed using CNVkit (0.9.1) that is available on the NIH Biowulf HPC cluster. To build the reference before copy number calling, we added –method wgs. In addition to default parameters, the parameter –center-at -0.51 has been used, where -0.51 is equivalent to log2 ( 2 / 2.85). 2 / 2.85 is the ratio between the ploidy assumed by CNVkit (2) and the ploidy reported in the literature for the HCC1395 cell line (2.85 [23]). The log2 of the ratio manually re-centers the calls by shifting them in log-scale.

### FACETS

Samples (WGS, WES, SPP, LBP, FFX, FFG) of SEQC-II triple-negative breast cancer cell line HCC1395 were analyzed using FACETS (0.6.0), available on the NIH Biowulf HPC cluster. FACETS was run using the default parameters or parameters recommended by the user’s manual with hg38 reference genome. For FACETS, we used “htstools/snp-pileup” and set FACETS cval = 50 for “procSample”.

#### Control-FREEC (WGS)

Control-FREEC (v11.6) somatic CNV detection was used to process WGS tumor/normal samples per the recommendation and user guide of Control-FREEC. A config text file was created to include all the parameters and run the detection tool how it is intended to be run. There are many different parameters and options that can be set with Control-FREEC, but for the purpose of this study we decided to work with the most basic parameters to give an overall view of this CNV detection tool. The parameters are set up in the config file in different sections. The ‘General’ section takes parameters that will work with CNV detection and fine tune it to gather more predicted segments and accurate segments as well. The parameters we defined are described as follows. We defined the chrLenFile with a path to the.fai file which would have all the listed chromosome lengths. We specified the window of detection at 50,000 kb. The breakPointThreshold was set at 0.04 to predict more CNVs in the sample. We defined maxThreads to help speed up the process and run the tool over different threads. The path to all the chromosome files was provided as well as the output directory where we wanted all of the files to be placed.

The “sample” and “control” segments of the config file were identical except in the case of the bam file provided. The sample was defined as the tumor bam file and the normal was placed in the control section. This allowed us to run the tool sswith a tumor/matched normal pair. The resulting CNV file was then run through an R script that was also provided by the Control-FREEC documentation and this script the *p*-values for each CNV segment recorded.

Other running parameters: window = 50000 breakPointThreshold = 0.04 sex = XX maxThreads = 240 ploidy = 3.

For each sample, we computed the overall ploidy as the length-weighted average of total copy number, across the full genome, i.e., each called segment was considered towards a total sum (across all segments) by adding as addend the product (segment length * total copy number of the segment). The total sum of all segments’ products was then divided by the total length of the genome covered by called segments.

#### Control-FREEC (WES)

To run Control-FREEC for WES samples, another config text file was created, similar to that for running on WGS samples. There was one different parameter that was used to run Control-FREEC for WES samples and that was the addition of a target region and a bed file with those regions specified. The window parameter was also taken out, whereas for WGS samples the window size was set to 50,000.

#### HATCHet

HATCHet (v1.0.4) [17] somatic CNV detection was used to process WGS tumor/normal samples per the recommendation and user guide of HATCHet. HATCHet was an algorithm that used a Pyomo solver to find copy number aberrations in tumor samples. HATCHet can handle bulk tumor samples at once matched to normal sample, but for the purpose of our study we only ran HATCHet on a single tumor/matched normal sample at a time. The Pyomo solver recommended for use was Gurobi and a Gurobi license was obtained for this study.

HATCHet has many different steps within it and each step has parameters that can be changed. Our runs did not use the plotting steps “plot bins” and “plot cn,” and only focused on getting the CNV counts. Under “count reads” we set the vin size to 50 kb to be consistent with the window size we used in Control-FREEC, which differs from the bin size of 200 kb recommended by HATCHet for WES samples. Under “genotype snps” the reference version for the germline SNPs must be specified (hg38 for our study). A path to a SNP list does not have to be defined as HATCHet will generate one based on the data given. Default parameters for “compute cn” were used. The clones parameter generated the number specified and would output the best solution and generated CNVs. An example file containing run parameters can be found in the Supplementary Material.

#### ascatNgs

ascatNgs (v2.5.1) [13] somatic CNV was used to process WGS T/N samples per recommendations from the ascatNgs user guide. ascatNgs another software tool that detects CNV with tumor matched normal samples. It can also estimate tumor purity and ploidy. The execution of ascatNgs is simpler than the other methods listed before it. Only a command line entry is needed for this tool to run. The parameters needed are the reference file, tumor, and normal bam files to start. Then a GC correction tsv file is needed, this was obtained from the ascatNgs github page to remain consistent. The last few parameters needed are just specifications of the file and genome type for ascat to run and predict the CNVs accurately: WGS vs WES, gender, species, and reference assembly. The parameters used in our command line were all required for the pipeline to run.

##### DRAGEN

DRAGEN somatic CNV was used to process WGS T/N samples per recommendations from the DRAGEN user guide. A custom build based on DRAGEN 4.0.x was used for this analysis with the following non-default parameters. A full example command line can be found in the Supplementary Material.

Target counts interval width: cnv-interval-width defaulting to 1000, except for lower coverage samples as mentioned below:

SPP 10 × where a value of 4000 was set.

LBP TruSeq Nano 10 ng where a value of 2000 was set.

LBP TruSeq Nano 1 ng where a value of 10,000 was set.

Merge distance: cnv-merge-distance set to 2,000,000.

Filter length: cnv-filter-length set to 50,000.

Exclusion BED: cnv-exclude-bed =  < BED > , indicating regions to exclude for analysis (6p, 16q, X).

To process WES T/N samples, DRAGEN somatic CNV was used per recommendations from the DRAGEN user guide. A custom build based on DRAGEN 4.0.x was used for this analysis, leveraging the Panel of Normals technique. A more robust purity/ploidy model is available for future versions of DRAGEN. The matched normal was first used as a single sample panel of normal to generate target.counts. The matched normal sample was then used in CNV calling, with the following non-default parameters:

CNV ploidy: cnv-ploidy 3.

CNV GC bias correction: cnv-enable-gcbias-correction false.

Filter quality: cnv-filter-qual 20.

Exclusion BED: cnv-exclude-bed =  < BED > , indicating regions to exclude for analysis (6p, 16q, X).

A full example command line is below:

dragen \

–output-directory = ${OUTPUT_DIRECTORY} \

–output-file-prefix = ${PREFIX} \

–ref-dir = ${REFERENCE} \

–bam-input = ${TUMOR_BAM} \

–cnv-exclude-bed = ${EXCLUDE_BED} \

–cnv-enable-ref-calls = true \

–cnv-target-bed = ${TARGET_BED} \

–cnv-normals-file = ${MATCHED_NORMAL_COUNTS} \

–cnv-enable-gcbias-correction = false \

–cnv-ploidy = 3 \

–cnv-filter-qual = 20 \

–enable-cnv = true

### Somatic CNV detection with other orthogonal technologies

#### Affymetrix Cytoscan HD microarray

We obtained DNA of two reference cell lines from ATCC (HCC1395, SCCRL2324 D; HCC1395 BL, SCCRL2325 D). DNA concentration was measured spectrophotometrically using a Nanodrop (Life technology), and integrity was evaluated with a TapeStation 4200 (Agilent). Two hundred and fifty nanograms of gDNA was used to proceed with the Affymetrix CytoScan Assay kit (Affymetrix). The workflow consisted of restriction enzyme digestion with Nsp I, ligation, PCR, purification, fragmentation, and end labeling. DNA was then hybridized for 16 h at 50 °C on a CytoScan array (Affymetrix), washed and stained in the Affymetrix Fluidics Station 450 (Affymetrix), and then scanned with the Affymetrix GeneChip Scanner 3000 G7 (Affymetrix). Data were processed with ChAS software (version 3.3). Array-specific annotation (NetAffx annotation release 36, built with human hg38 annotation) was used in the analysis workflow module of ChAS. Karyoview plot and segments data were generated with default parameters.

#### Bionano

Ultra-high molecular weight (UHMW) DNA was extracted from cryopreserved cells in frozen medium containing DMSO following the manufacturer’s protocols (Bio- nano Genomics, USA). Cells were digested with Proteinase K and RNAse A in a lysis binding buffer containing detergents. DNA was precipitated with isopropanol and bound with nanobind magnetic disk. Bound UHMW DNA was resuspended in the elution buffer and quantified with Qubit dsDNA assay kits (Thermo Fisher Scientific). DNA labeling was performed following the manufacturer’s protocols (Bionano Genomics, 188 USA). Standard Direct Labeling Enzyme 1 (DLE-1) reactions were carried out using 750 ng of purified ultra-high molecular weight DNA. The fluorescently labeled DNA molecules were counter-stained and imaged across nanochannels on a 2nd generation Saphyr instrument. Genome coverage of approximately 400X was achieved for all tested samples.

Genome analysis was performed using software provided by Bionano Genomics (Bionano Solve 3.5, Access 1.5). Specifically, the fractional copy number analysis was done for this current study. The fractional copy number analysis was performed from alignment of molecules and labels against GRCh38 (alignmolvref). A sample’s raw label coverage was normalized against relative coverage from normal human controls, segmented, and baseline CN state estimated from calculating mode of coverage of all labels. If Y chromosome molecules were present, baseline coverage in sex chromosomes was halved. With a baseline estimated, CN states of segmented genome intervals were assessed for significant increase/decrease from the baseline.

Corresponding duplication and deletion copy number variant calls were output. Certain SV and CN calls were masked, if occurring in GRC38 regions found to be high variance (gaps, segmental duplications, etc.)

CNV calling was done on three replicates of the breast cancer sample HCC1395 and the paired control sample HCC1395BL. A non-redundant CNV set was generated for the two samples using a custom clustering algorithm. The clustering algorithm merges CNVs of the same type, in proximity (within 10 kbp position) and with a similar size (> 50% size similarity).

#### Illumina Infinium CytoSNP-850 K v1.3 BeadChip

Reference cell lines HCC1395 BL and HCC1395 were analyzed in triplicates on CytoSNP-850 K v1.3 BeadChips following reference guide (https://support.illumina.com/content/dam/illumina-support/documents/documentation/chemistry_documentation/infinium_assays/infinium_cytosnp-850k/infinium-cytosnp-850k-reference-guide-1504.pdf.). The array signals were captured with iScanTM Array Scanner. Data for all samples were processed in GenomeStudio (version 2.0.5) using the cnvPartition algorithm (version 3.2.1) with GC wave adjustment. CNV calls with minimum support of 3 probes and confidence score above 35 were retained. The CytoSNP-850Kv1- 1 iScan C2 (hg38) version of the manifest and cluster file were used for the analysis.

### Reproducibility calculation

There are three types of variants in the original CNV call set, including gain calls, loss calls, and LOH calls. The copy number cutoff values are set as follows: for gain calls, the copy number is greater than 2; for loss calls, the copy number is less than 2; for LOH calls, the copy number is equal to 2. We divided the original CNV call set into gain calls, loss calls, and LOH calls, then computed their reproducibility separately. The Jaccard index was utilized to evaluate the reproducibility or similarity of two scall sets. It is calculated as the intersection of the length of genomic regions of CNV calls between two call sets divided by the union length. The Jaccard index was computed using the “jaccard” command of BedTools [25]. CNVs on the p-arm of chr6, q-arm of chr16, chrX, and gap regions (including centromere regions and telomere regions) were excluded from this analysis. The exclusion of the p-arm of chr6, q-arm of chr16, and chrX is based on our previous study [10], where we confirmed that these regions are entirely lost in the genome of HCC1395. The exclusion of gap regions is attributed to the difficulties associated with accurately calling CNVs due to misalignment of NGS short reads. When assessing the reproducibility of WGS call sets at the segment level, all CNV segments on the whole genome were included. In evaluations at the gene and exon levels, CNV-affected regions reproducibility within annotated gene or exon regions by Refseq were retained. For comparisons of reproducibility between WGS and WES datasets, CNV-affected regions within exons annotated by Refseq were considered.

### Partitioning, scoring, and assigning confidence levels for CNV intervals

A total of 21 fresh tumor and matched normal WGS replicates were generated, with distribution across different centers: FD (3 replicates), IL (3 replicates), NV (3 replicates), NS (9 replicates), EA (1 replicate), NC (1 replicate), and LL (1 replicate). Each replicate underwent analysis using six CNV callers, yielding a total 126 call sets. We divided the original CNV call set into gain calls, loss calls, and LOH calls, to integrate their benchmark calls separately. The representations, including length and breakpoints, of the same CNVs typically varied among these call sets. We utilized the “multiinter” command from BedTools to partition all overlapping regions into a set of disjoint intervals. These intervals were segmented based on the number of supported call sets (Additional file 1: Fig. S4B).

The confidence levels for each CNV interval were assessed based on reproducibility at three levels: within lab, cross lab, and cross callers. A group score is assigned based on reproducibility within the lab and cross lab. Following that, confidence levels are assigned based on the reproducibility across callers. Five group scores were initially assigned, considering reproducibility across 21 replicates from six sequencing centers for each of the six callers. To obtain a group score, the 21 replicates were divided into five groups for each caller, as illustrated in Fig. S4A. Replicates from the same center are expected to yield consistent results. We first assessed reproducibility among replicates within the same center. Consequently, three replicates from FD were grouped as Group 1, three replicates from IL as Group 2, three replicates from NV as Group 3, nine replicates from NS as Group 4, and one replicate each from EA, LL, and NC were designed as Group 5. A CNV interval received an intermediate score based on the number of supported replicates within each group, ranging from 0 to 3. For example, in a group with three replicates from a specific caller, is CNVs are called by call set 1 and 2 but not 3, the intermediate score be- fore being added up as a group score is 2. In Group 4, consisting of nine replicates from NS, their intermediate score was divided by 3. The group scores for each caller were then aggregated across the five groups, and the cumulative group score was converted to a range of 0 to 3: 10–15 to 3, 7–9 to 2, 4–6 to 1, 0–3 to 0. A lower group score indicated less concordance among replicates and sequencing centers (Additional file 1: Fig. S5A). Subsequently, we assigned one of four confidence levels (strong evidence, medium evidence, weak evidence, and neutral evidence) to each CNV interval based on the sum of five group scores from the six callers (Additional file 1: Fig. S4B). The conversion scale was as follows: 11–18 to strong evidence, 8–10 to medium evidence, 4–7 to weak evidence, and 1–3 to neutral evidence. A descending scale in scoring and evidence indicated calls found in fewer replicates and by fewer callers. Only five callers detected LOH: CNVkit, Control-FREEC, DRAGEN, FACETS, and HATCHet, resulting in 105 call sets (21 replicates × 5 callers). Following the same process used to integrate high-confidence copy number gains and losses, we first partitioned the overlapping LOH segments in to small disjointed intervals. Subsequently, we integrated group scores for each of the five callers based on the reproducibility across replicates and centers (Additional file 1: Fig. S4A). We then assigned one of four confidence levels to each cluster (strong, medium, weak, and neutral) based on the reproducibility of callers (Additional file 1: Fig. S6). LOH regions with an aggregated group score greater than 8 were classified as strong evidence calls, while medium evidence calls encompasses those with an aggregated group score between 6 and 7. Weak evidence calls included regions with an aggregated group score between 4 and 5, and neutral evidence calls comprised regions with an aggregated group score less than 3.

### Calculation of precision, recall, and F1 score

Precision, recall, and F1 score were calculated by comparing the set of CNV calls with high-confident calls defined in this study. Precision is the fraction of CNV-affected regions of a query set which are the same with high-confident calls’ regions. Recall is the fraction of CNV-affected regions of high-confidence calls which are called by a query set. F1 score is the harmonic mean of the precision and recall. The comparison was done using BEDTools’s jaccard command.

### Supplementary Information

Additional file 1. Figures_Tables, supplementary figures, tables, and methods.

Additional file 2. CNV_genelist, genes in the CNV affected regions.

Additional file 3. cnv_gain_cn_median, copy number calls in gain regions.

Additional file 4. cnv_loss_cn_median, copy number calls in loss regions.

Additional file 5. cnv_benchmark_calls, high confidence CNV call set in vcf format.

Additional file 6. Review history.

## Data Availability

All data (bam files) used in this study were downloaded from NCBI’s ftp site: https://ftp-trace.ncbi.nlm.nih.gov/ReferenceSamples/seqc/Somatic_Mutation_WG/data/. Raw data for Illumina CytoSNP-850 K array is available on NCBI GEO database [26]. CNV benchmark sets and scripts used for statistical analysis are available at Zenodo [27, 28].
