# Supplementary material for: Evaluation of somatic copy number variation detection by NGS technologies and bioinformatics tools on a hyper-diploid cancer genome
Source: Genome Biol. 2024 Jun 20;25:163. doi: 10.1186/s13059-024-03294-8 (PMC11188507; doi:10.1186/s13059-024-03294-8)
Supplement: Supplementary file 1 — Additional file 1. Figures_Tables, supplementary figures, tables, and methods. [file 13059_2024_3294_MOESM1_ESM.docx]

Supplementary Material for

Evaluation of somatic copy number variation detection by NGS technologies and bioinformatics tools on a hyper-diploid cancer genome

**Contents**

[Supplementary Figures](#_bookmark0) 2

[Supplementary Tables](#_bookmark1) 15

[Supplementary Methods](#_bookmark2) 17

# Supplementary Figures

**


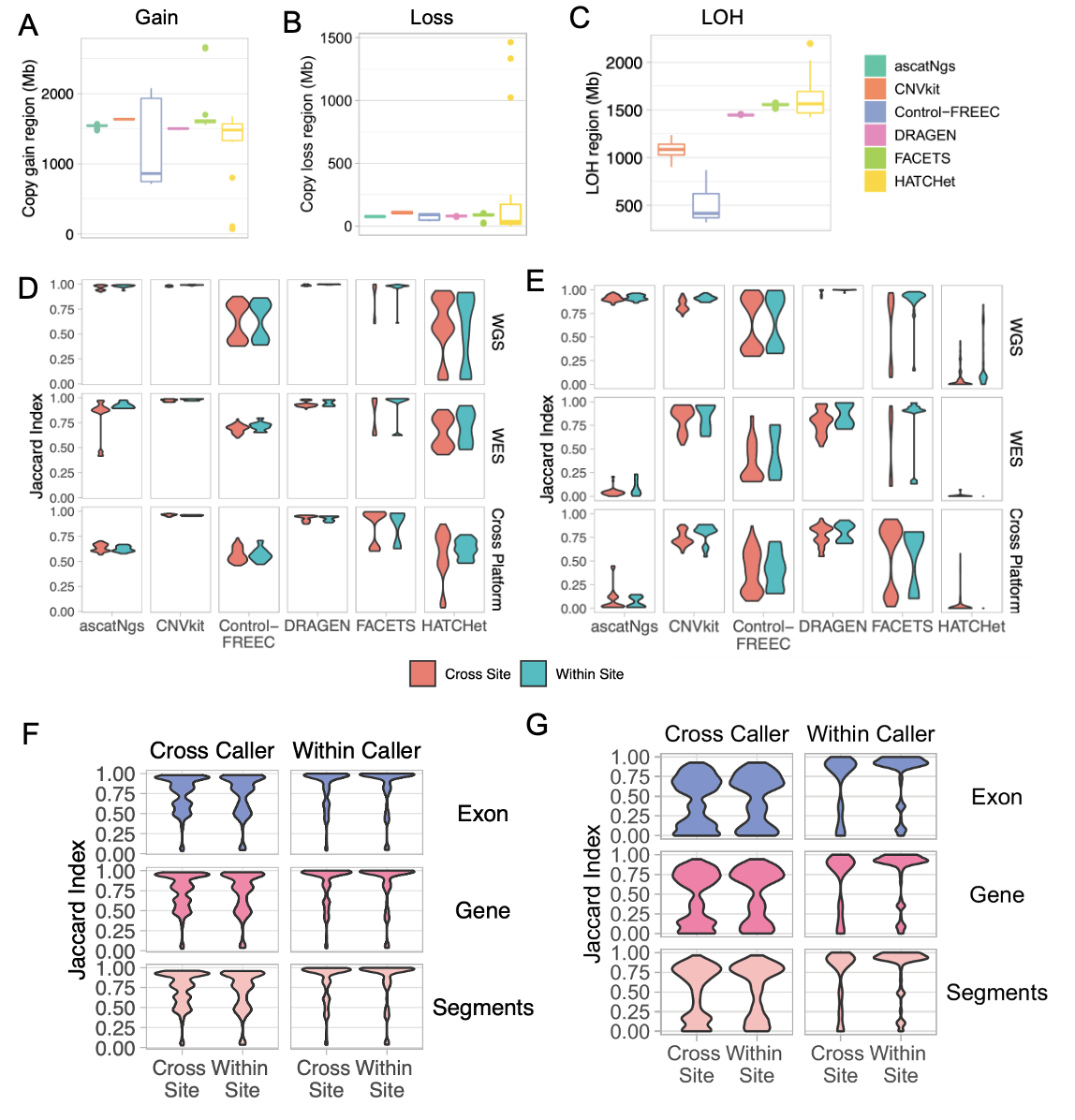
**

Fig. S1: (A) Genome regions with gain calls by each caller in 21 WGS replicates. (B) Genome regions with loss calls by each caller in 21 WGS replicates. (C) Genome regions with loss calls by each caller in 21 WGS replicates. Violin plots of the concordance of genomic regions with gain (D) or loss (E) calls in WGS and WES replicates across six callers. (F) Concordance, measured by Jaccard index, of segments, gene regions, and exon regions. (G) Concordance, measured by Jaccard index, of segments, gene regions, and exon regions


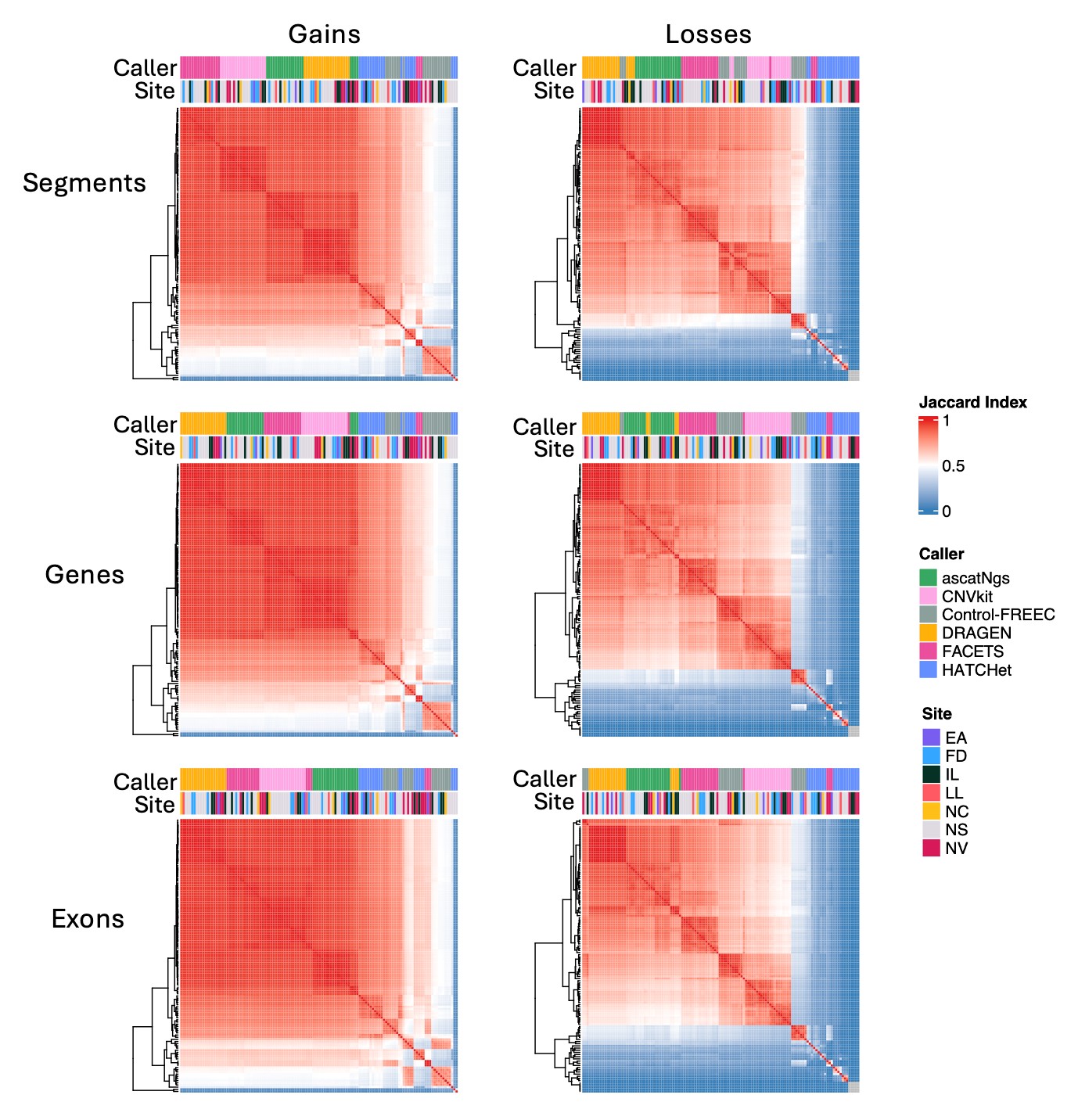


Fig. S2: Concordance, measured by Jaccard index, of genome regions (segments), gene regions, and exon regions for 21 WGS replicates. The color of each cell in the heatmap represents the Jaccard index, where high similarity if denoted by red and low similarity by blue, calculated based on the length of shared CNV regions divided by the union of CNV regions from two call sets.


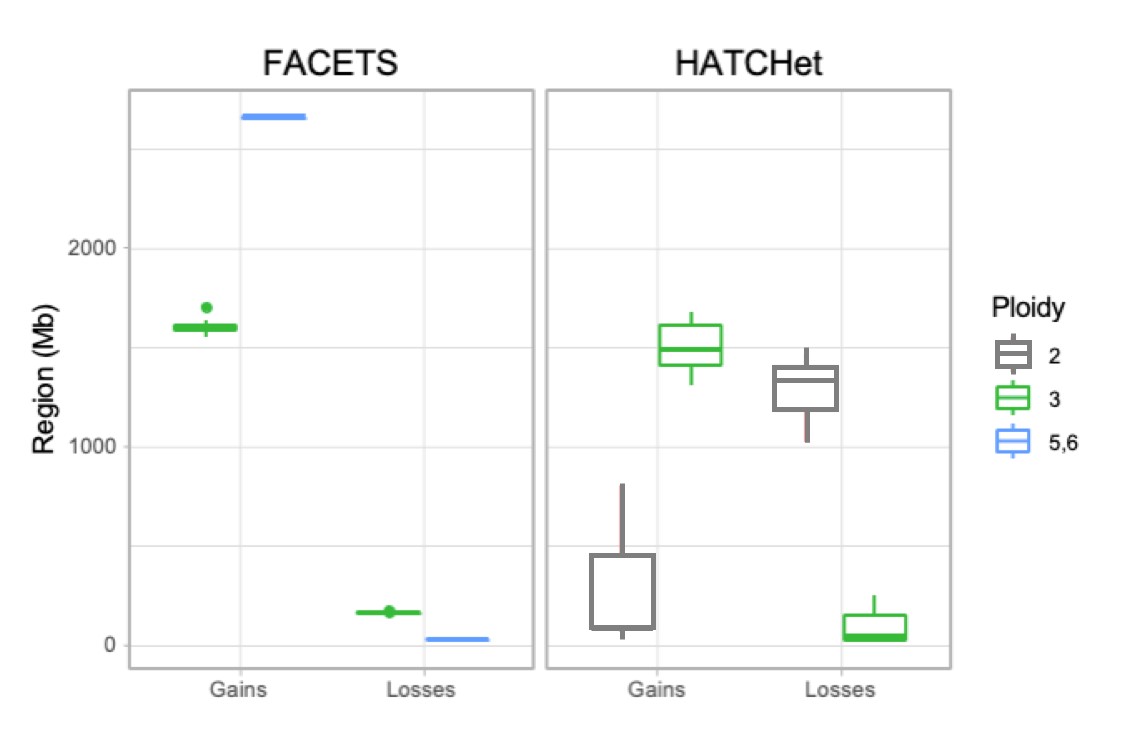


Fig. S3: Total genomic regions of gains and losses called by FACETS and HATCHet on the WGS dataset stratified by ploidy assessed by caller on each replicate. Note, a correct ploidy assessment should be 3.


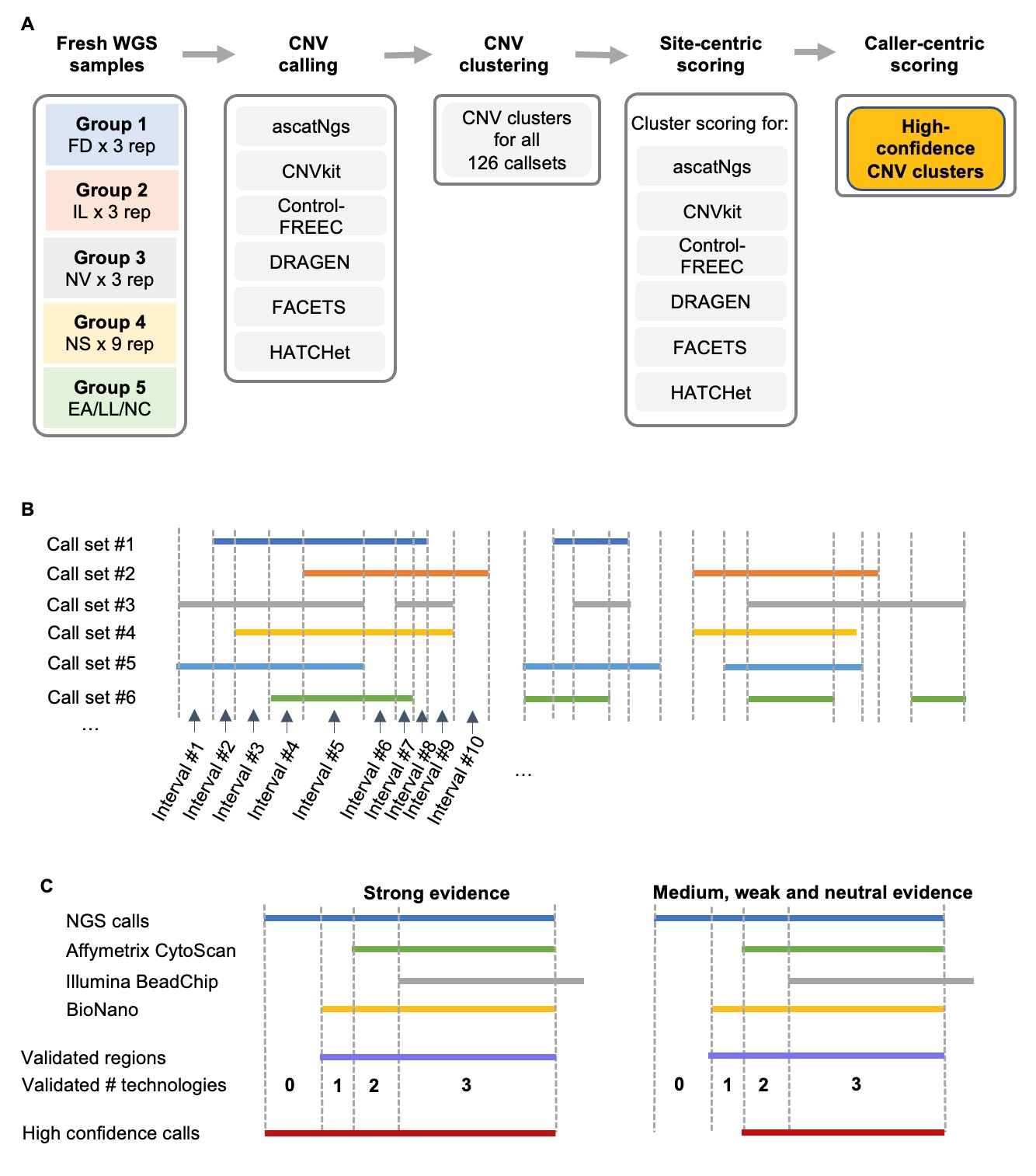


Fig. S4: Schematic diagram of integration and validation of CNV benchmark sets. (A) Overall, 21 WGS replicates were grouped into 5 groups. Site 1: three replicates from Fudan; site 2: three replicates from Illumina; site 3: three replicates from Novartis; site 4: nine replicates from Illumina with NovaSeq; site 5: three replicates from EATRIS, Loma Linda, and NCI. Six CNV callers, ascatNgs, cnvkit, Control-FREEC, DRAGEN, FACETS, and HATCHet were used to generate a total of 126 call sets where CNV clusters were derived. (B) Schematic diagram of CNV interval partition. (C) Schematic diagram of NGS consensus CNV validation by other three orthogonal technologies.


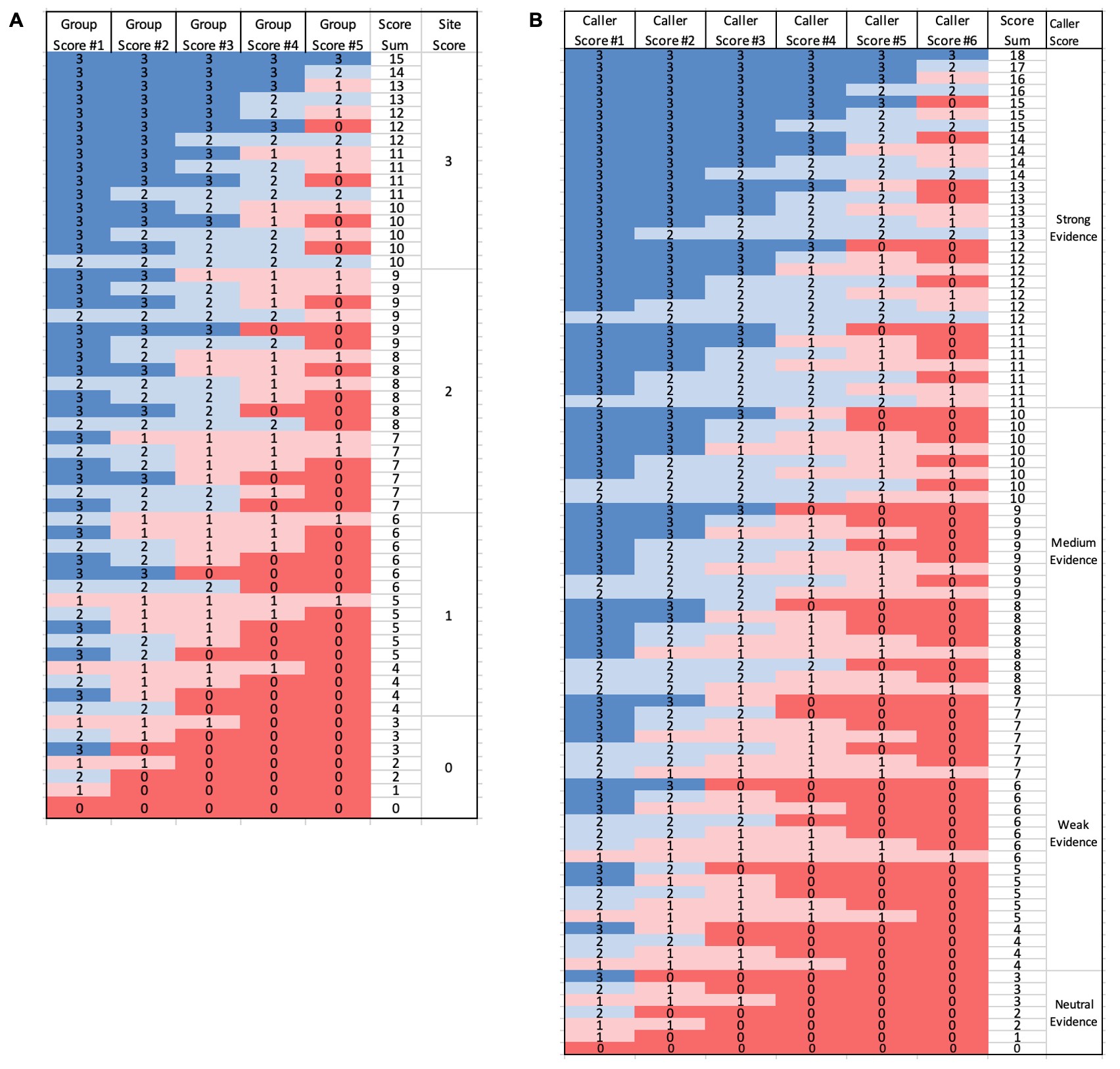


Fig. S5: CNV gain and loss interval scoring scheme per site replicates and consensus across six callers. (A) Intermediate Scoring system of 21 callsets for site score for each caller. (B) Intermediate Scoring system of 6 integrated callsets for confidence level for each caller.


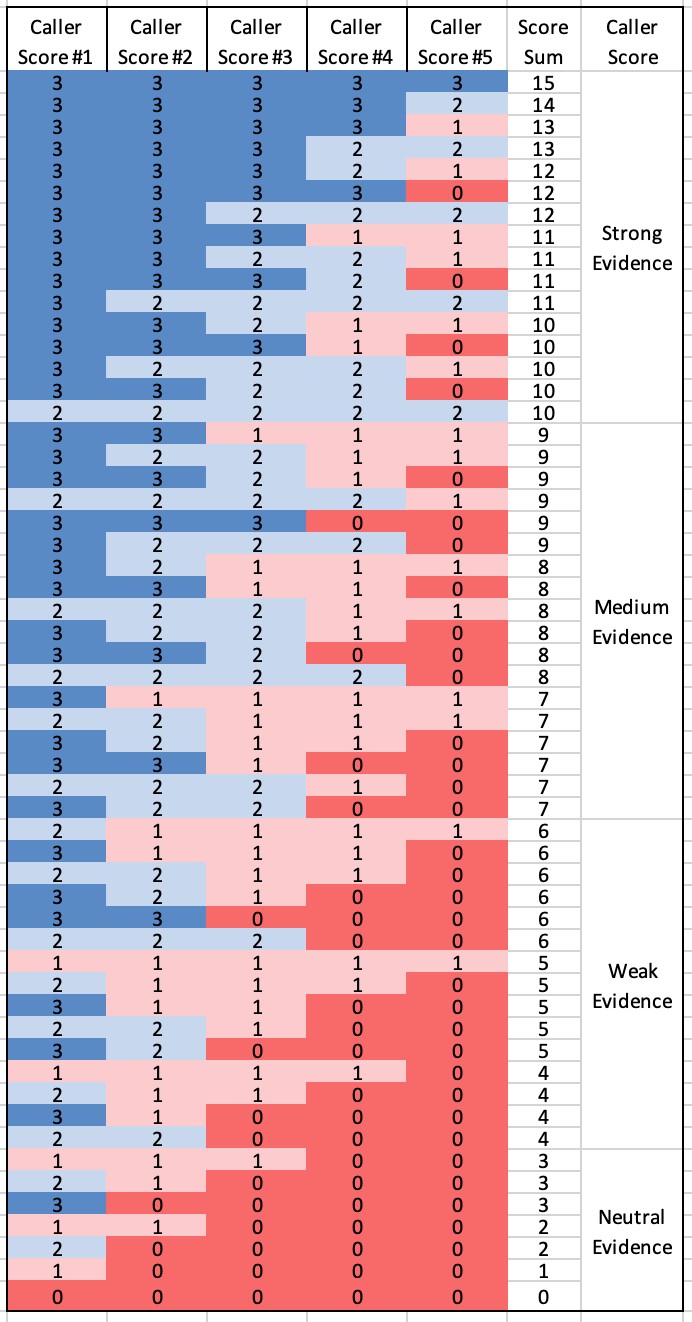


Fig. S6: CNV LOH interval scoring scheme for confidence levels across six callers.


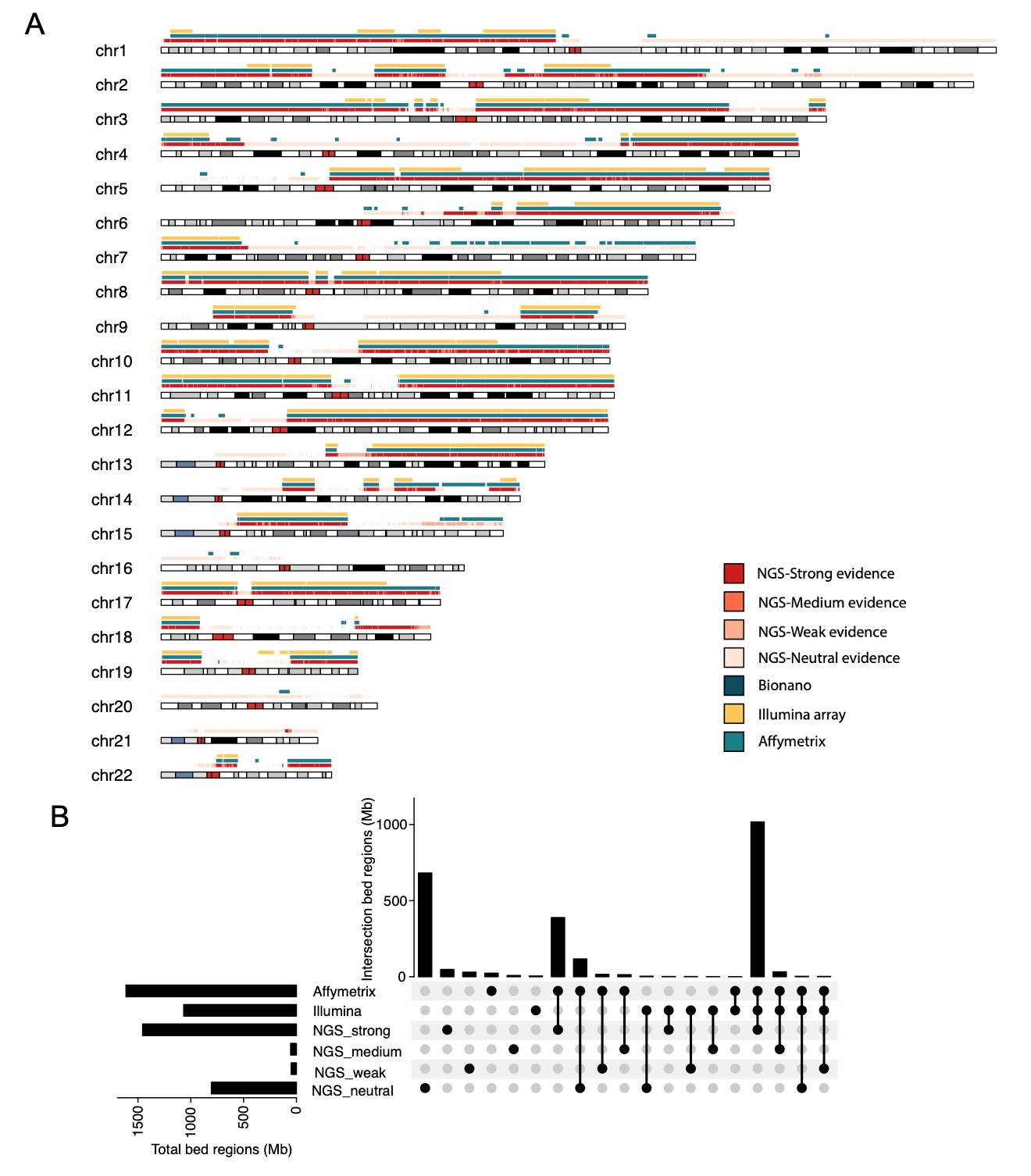


Fig. S7: Comparison of LOH calls by CNVkit, Control-FREEC, DRAGEN, FACETS, and HATCHet to results from microarray. (A) Chromosome view of LOH clusters in comparison to results from microarray.

(B) Upset plot of concordance of LOH cluster with different confidence level in comparison to results from microarray.


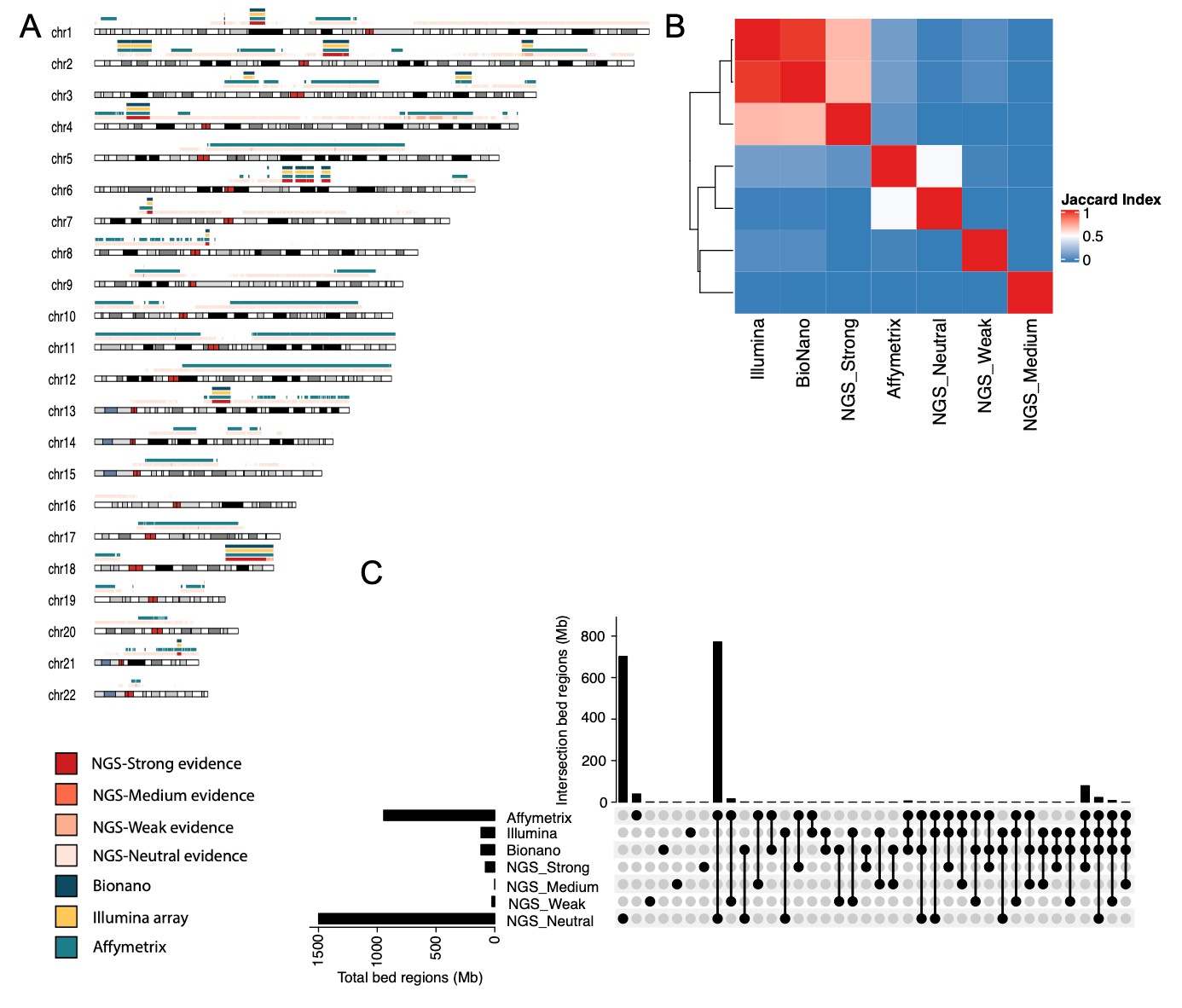


Fig. S8: Consistency of collapsed loss clusters in comparison to three orthogonal methods. (A) Chromosome view of loss clusters in comparison to three orthogonal methods. (B) Concordance of Jaccard index scores for loss cluster with different confidence level in comparison to three orthogonal methods. (C) Upset plot of concordance of loss cluster with different confidence level in comparison to three orthogonal methods.


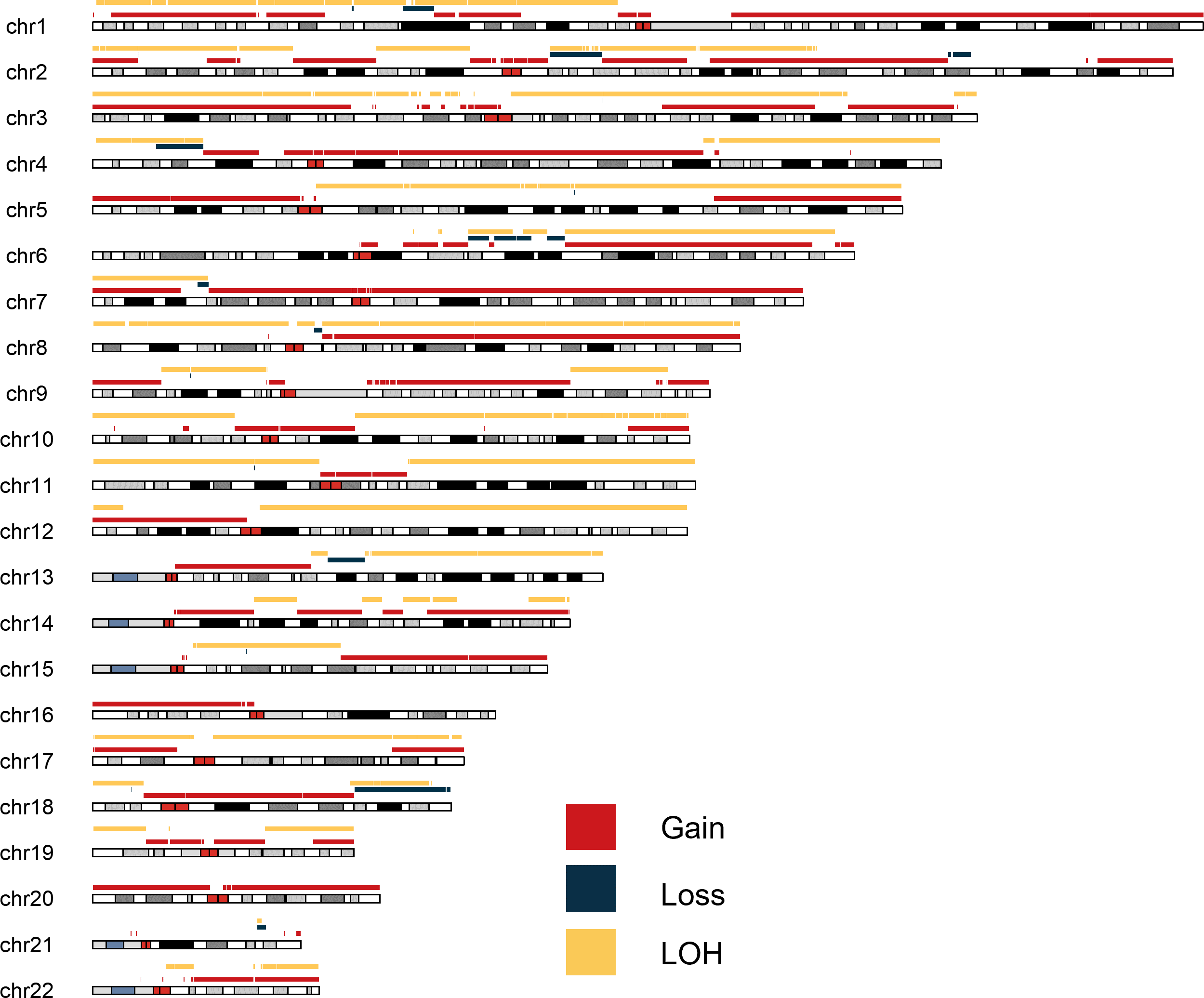


Fig. S9: Chromosome view of genome regions in the high confidence call set gain, loss, and LOH.


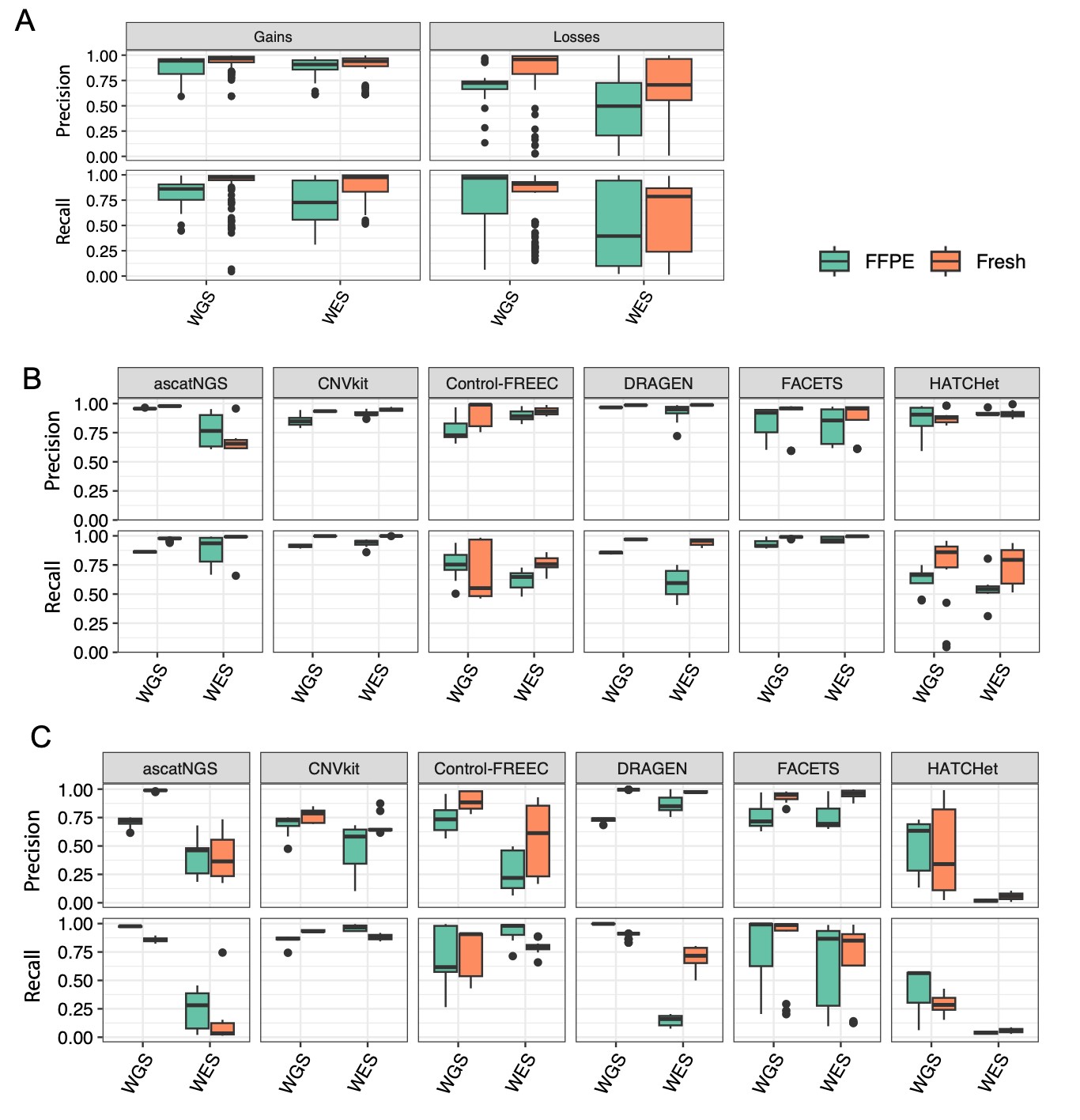


Fig. S10: Precision and recall of copy number calling methods across fresh and FFPE samples sequenced by whole-genome and whole-exome sequencing. A) Overall precision and recall across all copy number calling methods. B) Precision and recall results for gains further stratified by copy number calling method. C) Precision and recall results for losses further stratified by copy number calling method.


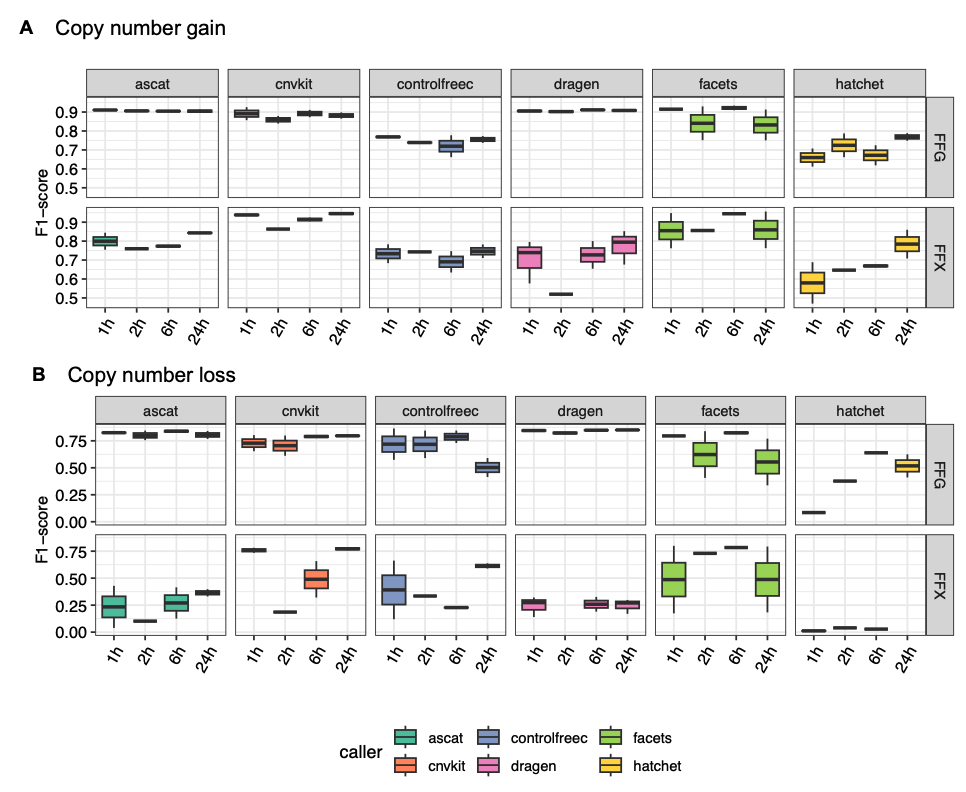


Fig. S11. The F1 score for copy number gain (A) and copy number loss (B) under varying FFPE fixing time.


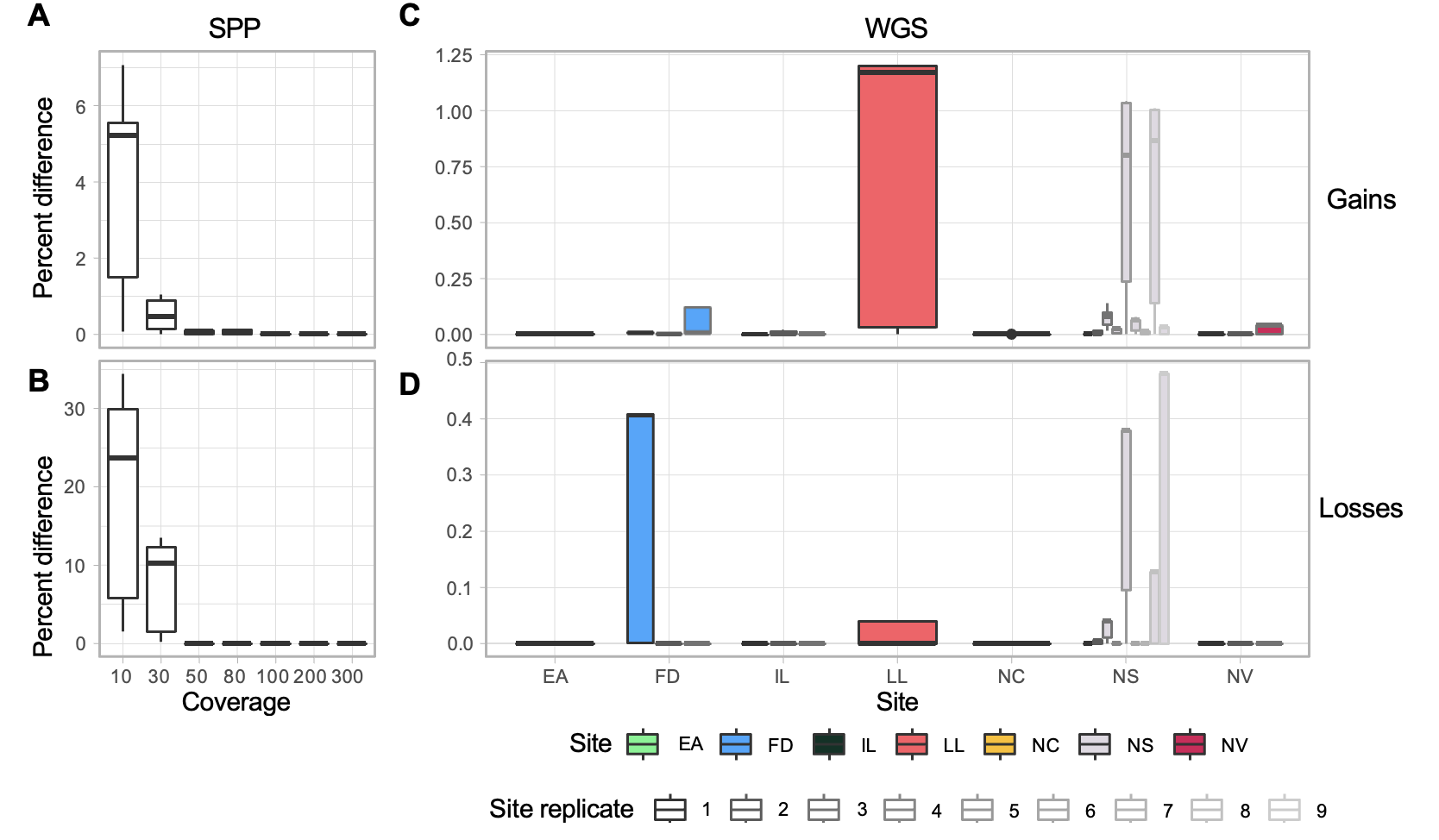


Fig. S12: Variability of ascatNgs. Boxplots of percent difference of gain and loss regions between runs of individual samples in the SPP dataset (A and B) and WGS replicates (C and D). Y-axis for C and D, percent of difference.

# Supplementary Tables

| WGS runs | Computed Ploidy by Control-FREEC |
| --- | --- |
| WGS_EA_1 | 5 |
| WGS_FD_1 | 3 |
| WGS_FD_2 | 5 |
| WGS_FD_3 | 5 |
| WGS_IL_1 | 3 |
| WGS_IL_2 | 3 |
| WGS_IL_3 | 3 |
| WGS_LL_1 | 5 |
| WGS_NC_1 | 3 |
| WGS_NS_1 | 3 |
| WGS_NS_2 | 5 |
| WGS_NS_3 | 3 |
| WGS_NS_4 | 3 |
| WGS_NS_5 | 5 |
| WGS_NS_6 | 3 |
| WGS_NS_7 | 3 |
| WGS_NS_8 | 5 |
| WGS_NS_9 | 3 |
| WGS_NV_1 | 3 |
| WGS_NV_2 | 3 |
| WGS_NV_3 | 3 |

Table S1: Computed ploidy estimate based on CNV calling results from Control-FREEC in each of WGS run.

|  | Number | Genomic Region (Mb) |
| --- | --- | --- |
| Gain | 340 | 1518.5 |
| Loss | 33 | 79.7 |
| LOH | 315 | 1456.1 |

Table S2: Genome regions and number of segments in the high confidence call set for gain, loss, and LOH.

|  | WGS | SPP |
| --- | --- | --- |
| Overall (gains and losses) | -0.26 | -0.35 |
| Gains | -0.26 | -0.39 |
| Losses | -0.32 | -0.44 |

Table S3: Correlation between coverage and percent difference in runs of ascatNgs on WGS and SPP datasets.

| Caller | Key parameters |
| --- | --- |
| FACETS | set.seed(1234), cval=50, res=300 |
| Control-FREEC | window=50000, breakPointThreshold = 0.04, maxThreads=40, mateOrientation = 0 [control] mateOrientation = 0 |
| HATCHet | Samples size = 50kb mincov = 8 100x maxcov = 300 mincov = 8 maxcov = 300 size= 50kb diploidbaf = 0.08 tolerancerdr = 0.15 tolerancebaf = 0.04 sizethreshold = 0.01 figsize = "6,3" clones = 2,6 seeds = 400 minprop = 0.03 diploidcmax = 6 tetraploidcmax = 12 ghostprop = 0.35 limitinc = 0.6 |
| ascatNgs | - ’reference.fa’ -t ’tumor bam’ -n ’normal bam’ -pr WGS -g XX -sg ’GC |
| DRAGEN | --cnv-merge-distance=2000000 \ --cnv-filter-length=50000 \ --enable-variant-caller=true \ --cnv-interval-width=${CNV_INTERVAL_WIDTH} \ --cnv-exclude-bed=${EXCLUDE_BED} \ --tumor-bam-input=${TUMOR_BAM} \ --bam-input=${NORMAL_BAM} \ --ref-dir=${REFERENCE} \ --sample-sex=female \ --enable-map-align=false \ --enable-cnv=true |
| CNVkit | --center-at -0.51 |

Table S4: Key parameters for six CNV callers.

# Supplementary Methods

### Example run commands for somatic CNV detection algorithms

FACETS

The following command line was used to run "htstools/snp-pileup" prior to FACETS:

htstools/snp-pileup 00-commo_all.vcf /outdir 1.bam 2.bam

An example script used to run FACETS is below:

library(facets) name="sampleid" set.seed(1234) fin=name

fout=paste("/outdir/sampleid_fit_cncf.txt",sep="") fstat=paste("sampleid_statistics.txt",sep="") img=paste("sampleid_plot.png",sep="")

df = readSnpMatrix(fin) xx=preProcSample(df, gbuild=c("hg38")) oo=procSample(xx,cval=50)

oo$dipLogR fit=emcncf(oo) head(fit$cncf)

cat("purity ",file=fstat,sep=" ") cat(fit$purity,file=fstat,sep=" ",append=TRUE) cat("ploidy ",file=fstat,sep=" ", append=TRUE) cat(fit$ploidy,file=fstat,sep=" ",append=TRUE) cat("dipLogR ",file=fstat,sep=" ", append=TRUE) cat(oo$dipLogR,file=fstat,sep=" ",append=TRUE)

write.table(fit$cncf, file = fout, sep = " ",row.names = TRUE, col.names = TRUE) png(img, width=8, height=6, units="in", res=300)

plotSample(x=oo,emfit=fit) dev.off()

Control-FREEC (WGS

The command line for running Control-FREEC:

freec -conf $<$config file$>$

An example of the config file that was used is below:

[general]

chrLenFile = /home/daniall.masood/reference/GRCh38/GRCh38.d1.vd1.fa.fai window=50000

breakPointThreshold = 0.04 sex=XX

maxThreads=40

chrFiles = /home/daniall.masood/GRCh38 outputDir = /home/daniall.masood/test/WGS/

[sample]

mateFile = /home/daniall.masood/WGS/WGS_NV_T_1.bwa.dedup.bwa.dedup.bam inputFormat = BAM

mateOrientation = 0 [control]

mateFile = /home/daniall.masood/WGS/WGS_NV_N_1.bwa.dedup.bwa.dedup.bam inputFormat = BAM

mateOrientation = 0

HATCHet

An .ini file was created to include all the parameters and run the detection tool how it is intended to be run. The command line for running HACTHet: hatchet-run hatchet.ini An example of the .ini file is included below:

[run]

# Valid values are True or False count_reads = True

genotype_snps = False count_alleles = True combine_counts = True cluster_bins = True plot_bins = False compute_cn = True plot_cn = False

# Path to reference genome

# Make sure you have also generated the reference dictionary # as /path/to/reference.dict

reference = "/home/daniall.masood/GRCh38/GRCh38.d1.vd1.fa"

# Make sure you have generated the .bam.bai files at the same locations as these bam files normal = "/home/daniall.masood/SPP/SPP_GT_0-1_mergeThree.RG.bwa.RG.bwa.bam"

# Space-delimited list of tumor BAM locations

bams = "/home/daniall.masood/SPP_GT_3-1_mergeThree.RG.bwa.bam" # Space-delimited list of tumor names

samples = "Primary"

# Output path of the run script

output = "/home/daniall.masood/Hatchet/SPP_300X_75"

# How many cores to use for the end-end pipeline?

# This parameter, if specified, will override corresponding

# ’processes’ parameters in individual <step> sections below. processes = 10

[count_reads]

# Bin size for calculating RDR and BAF

# 50kb used for WGS samples and 200kb used for WES Samples size = 50kb

[genotype_snps]

# Reference version used to select list of known germline SNPs;

# Possible values are "hg19" or "hg38", or leave blank "" if you wish for all positions to be genotyped reference_version = "hg38"

# Does your reference name chromosomes with "chr" prefix?; True or False chr_notation = True

# Use 8 for WGS with >30x and 20 for WES with ~100x mincov = 8

# Use 300 for WGS with >30x and Use 1000 for WES with ~100x maxcov = 300

# Path to SNP list

# If blank, HATCHet selects a list of known germline SNPs based on <run.reference_version> and <run.c # If not, please provide full path to a locally stored list (.vcf.gz) here.

snps = ""

[count_alleles]

# Use 8 for WGS with >30x and 20 for WES with ~100x mincov = 8

# Use 300 for WGS with >30x and Use 1000 for WES with ~100x maxcov = 300

[combine_counts]

# Haplotype block size used for combining SNPs blocklength = 50kb

# Path to phased file; leave as "None" to run hatchet without phasing phase = "None"

[cluster_bins] #0.08

diploidbaf = 0.08

tolerancerdr = 0.15

tolerancebaf = 0.04

[plot_bins] sizethreshold = 0.01

figsize = "6,3"

[compute_cn] clones = 2,6

seeds = 400

minprop = 0.03

diploidcmax = 6

tetraploidcmax = 12

ghostprop = 0.35

limitinc = 0.6

ascatNgs

An example of the command used to run ascatNgs:

ascat.pl -o ’output folder’ -r ’reference.fa’ -t ’tumor bam’ -n ’normal bam’ -pr WGS -g XX -sg ’GC

DRAGEN

A full example command line for running DRAGEN is below:

dragen \

--output-directory=${OUTPUT_DIRECTORY} \

--output-file-prefix=${PREFIX} \

--cnv-merge-distance=2000000 \

--cnv-filter-length=50000 \

--enable-variant-caller=true \

--cnv-interval-width=${CNV_INTERVAL_WIDTH} \

--cnv-exclude-bed=${EXCLUDE_BED} \

--tumor-bam-input=${TUMOR_BAM} \

--bam-input=${NORMAL_BAM} \

--ref-dir=${REFERENCE} \

--sample-sex=female \

--enable-map-align=false \

--enable-cnv=true
