# Supplementary material for: Evaluation of somatic copy number variation detection by NGS technologies and bioinformatics tools on a hyper-diploid cancer genome
Source: Genome Biol. 2024 Jun 20;25:163. doi: 10.1186/s13059-024-03294-8 (PMC11188507; doi:10.1186/s13059-024-03294-8)
Supplement: Supplementary file 6 — Additional file 6. Review history. [file 13059_2024_3294_MOESM6_ESM.docx]

Review History

**First round of review**

**Reviewer 1**

**Were you able to assess all statistics in the manuscript, including the appropriateness of statistical tests used?**

There are no statistics in the manuscript.

**Comments to author:**

Summary:

This manuscript contributed two extremely useful things to the literate: 1) a CNV reference call set for a highly rearranged cancer cell line and 2) an evaluation of popular CNV callers across a range of WGS and WES sequencing parameters.

The key fault in this manuscript is that it fails at 1) as the 'truth' set is just a consensus call set of the callers being compared thus all results are not based on whether a region has a CN change in the cell line has but a measure of the extent to which the CNV callers agree with each other.

CNVs and SVs are fundamentally intertwined semi-orthogonal measurements of the underlying genomic rearrangements. For every SV there needs to be either a compensating SV at the same location or a corresponding copy number transition at that location. Similarly, every (non-telomeric/end of chromosome) CN transition should have an explanatory SV. Understanding the complex genomic rearrangements in this sample (and cancer more generally) involves additional analysis (e.g. JaBba [PMC7912537]/LINX[PMC9903802]) the inputs to which is a consistent SV+CNV call set. That is, one should be able to construct a breakpoint graph.

The sample used here is particularly interesting from this perspective because it's a highly rearranged sample - the authors' consortia has previously published a high confidence SV truth set with 1788 events/breakpoints (c.f. the COLO829 somatic truth set with just 72 high confidence SVs). The authors state "We believe that the high-confident CNV call set on a hyper-diploid genome will be very useful for the cancer research community to benchmark existing tools or develop new algorithms with improved performance." and I completely agree with them. I look forward to having such a truth set on such an interest sample. Unfortunately, this manuscript does not provide such a truth set - there is a >2x discrepancy between the high confidence SV call set (1788 SVs) and this high confidence CNV call set (699 gain/loss/LOH segments).

Whilst this discrepancy can be partially explained by the purely depth-based CNV callers compared here being unable to detect small events (thus missing the short CN segments associate with them), this is more of a limitation of the callers chosen than anything fundamental. Callers such as CONSERTING, WEAVER and PURPLE utilise SV breakpoints in their segmentation algorithms and can provide base-pair accurate CN bounds. PURPLE is a particularly notable exclusion as not only is a recent tool used on a very large cohort (PMC9903802/) but the authors used the ploidy estimation from PURPLE in this manuscript but didn't include it in the comparison (at least for the WGS section - PURPLE does not appear to have a WES mode but, as you noted in your discussion, this isn't any different to ascat). This manuscript is completely missing any analysis of the accuracy/resolution of the CN callers, if/how this is improved at high depth (some callers have had parameters changed to reduce resolution at low depth). That is, the smallest segment size each caller can reliably report, and how far off the CN bounds are from the true CN transition/breakpoint position.

Furthermore, classifying merely as GAIN/LOSS/LOH is not sufficient for comprehensive somatic genomic rearrangement analysis. Complex events such as a double minutes, breakage-fusion-bridge and chromothripsis are common in many cancers and knowing whether a CN segment is CN 4, 8, or 100 is absolutely essential for differentiating BFB from double minutes.

The results outlined in this manuscript were not provided for review. Notably, I couldn't find the high confidence call set. As such, I am unable to determine whether some of the issues I raise below in the detailed comments are critical issues that materially impact results or are minor issues that can easily be corrected.

In conclusion, this manuscript does not provide the high confidence truth set it purports to, nor does it consider CN caller resolution. I look forward to a revised manuscript that provides the CNV component of the base-pair consistent SV+CNV truth set the cancer genomics bioinformatics research community absolutely needs.

Detailed comments:

P2 L29/P9 L14 the estimated ploidy of 2.85 uses the CNV caller PURPLE. Why wasn't GRIDSS2+PURPLE included in this benchmark?

P2 L38 How much of this difference is differences in inferred centromeric copy number? No current NGS CNV tools reliably centromeric CN. Some callers don't report centromeric CN and other calls infer a CN across them. Comparing the #bases of non-ref copy number across tools use these difference approaches is results in biased size counts. For a true like-for-like comparison, such regions should be excluded from analysis (and probably the acrocentric short arms as well as there's aren't many 'real' CN calls in these regions either). Does this difference in calling conventions explain the ~300Mb difference between AscatNgs/DRAGEN and CNVKit? I expect that all the analysis is impacted by this issue and the analysis should be repeated excluding these additional known problematic regions.

Fig 2B: what's driving the cross caller differences? Is it AscatNgs vs DRAGEN vs CNVKit clustering at the 0.75 peak? A caller vs caller breakdown would be more informative than just cross vs self. What's driving the inconsistent exons/genes for AscatNgs/DRAGEN/CNVKit? What about for segments? Are the segment differences driven by nested events or actual segmentation differences?

Fig S2: What are the site abbreviations? "Site" has not been mentioned prior to the point at which this figure is referenced in the main manuscript. It took me a while to realize that red = good and blue=bad - could you invert the colour scheme.

Fig2C: I don't know what this figure is presenting. There's no colour legend but it goes up to 126 so it looks like it's replicate count but it could be a cumulative segment count. The X axis is also label "Replicate" so it's really unclear what's being presented. All I can get from this is that ascatNgs/DRAGEN are the most consistent then CNVkit then FACETS and with Control-FREEC/HATCHet not great. What does "CNV Regions" mean and what's the colour actual represent? Please provide more clarity on this figure.

P4 L25: please provide a sentence or two in the main body about the meaning of your site score. I can't tell at this point whether a site score of 3 good or bad. See also my P13 L17 comment about the multiple meanings of "site" in this manuscript.

P5: See P13 comments regarding insufficient detail on how validation was performed and what was needed for a segment to be considered 'validated'. The high confidence call set would benefit considerably from validation of the CN transition locations with the long read data you already have.

P5 L17: The number of calls only makes sense in the context of consistency with your high confidence SV call set. How consistent are your CN transitions with your previously published SV call set? Note that this consistency can be used as a proxy for the joint quality of SV/CNV call sets (e.g. PMID: 3425323 Fig 3).

Once you include actual estimated CNs, you can further evaluate the consistency of the CNs. For example, if there's a CN transition from 3 to 1 at a position with an explanatory breakpoint, the other side of that breakpoint should also have a copy number delta of 2.

P5 L23 how is this validated? Your methods state you use bedtools multiinter to break up intervals but that's going to result in heaps of tiny clusters/segments at the start/end of each actual CN segment. Figure S10B indicates that you might perform some sort of merging, but it's not mentioned anywhere in the manuscript, and the unreferenced Fig S10B could also be an undocumented explanation of your Jaccard index calculation.

P5 L37 How much of the WGS/WES difference is driven by calling convention? Did you compare the genome-wide inferred CN from WES with WGS, or have you limited your WES vs WGS analysis to only the WES targeted regions? Generally speaking, the off-target WES CN isn't anywhere near as important as getting the exonic CN correct. What's the Jaccard index between WES and WGS in just the on-target regions?

P5 L52: what's driving this? Ploidy mis-estimation?

P5 L37: What is the gain concordance stratified by CN? Is the higher concordance of gains vs loss caused by some regions being highly amplified thus being really easy to call as gains? Are the CN=3 as good as CN=1? CN=4 the same as CN=0?

P6 L15: Does fixing time matter? Fig5 doesn't label the FFPE fixing time so I can't tell if there's a trend.

P6 L20: are you excluding regions that the CNV caller makes no call in your prec/recall calculations? NB: not all callers make a REF vs no-call distinction, but I know at least DRAGEN does if you give it the appropriate parameters.

P6 L35: How did you make >100x coverage data sets? From what I can tell, all replicates are less than 100x depth. Which samples and what merging logic did you use? Not really an issue for these callers, but if you add a SV+CNV pipeline such as GRIDSS2/PURPLE into the comparison, then merging sample with dissimilar fragment size distributions could be problematic on the SV calling side of things (I think you have enough similar replicates that this won't be an issue in practice).

P6 L38 could this performance plateau at 30x be converted into equal performance with a narrow window size thus an increased ability to detect small CN segments?

P6 L52: So are these variable HATCHet results intrinsic caller instability that is actually independent of variable under test? How similar are the HATCHet results if you take your 90x coverage replicates and randomly split the reads into three 30x non-overlapping subsets and run HATCHet? If there's variance in these three runs then it's likely that the instability is inherit in the HATCHet algorithm.

P7 L38. Please expand on ploidy estimate in the results section. Burying this important finding halfway through the discussion does disservice to just how important ploidy estimation is in somatic CNV calling. Ploidy estimation is a hard problem and even non-fold-change based approaches suffer from choosing purity/ploidy that half/double of the actual value.

P7 L57 With an actual ploidy results section, more of the ploidy discussion can be dedicated addressing various ploidy-based limitation and their practical impacts on real pipelines. E.g. do real-world CNVKit-based CN pipelines need to use PURPLE (or an equivalent tool) for purity/ploidy estimation like you have done here? Are ploidy estimates less reliable for WES vs WGS? (some of these questions request an entire cohort to comprehensively address).

P9 Not really needed in this manuscript but it would be useful to users if tool authors could update their documentation/default settings to reflect any non-standard best-practice parameters used in this comparison.

P13 L17. It is here that I find site means the sequencing centre performing the sequencing. Until this point I thought it was some classification of genomic site (e.g. STR, VNTR) and I just didn't know what the abbreviations were. Using something like "Sequencing Center" (like you do at P4 L32) would have been less confusing for me. Also, I still don't know what FD IL NV NS EA NV & LL actually mean.

P13 L27 "reprehensibility". Did you mean reproducibility?

P13 L30 I still don't understand how site score is calculated. Why split into 5 groups? Why are the groups named SUPP #1 to SUPP #5? Why did group 4 have 9 NS replicates? How does the method handle missing data (regions not called with any CN by a particular caller)? (Edit: I found meaningful group labels in Fig S10).

Given that your results indicate that sequencing centre doesn't really matter for the results, what's the value in retaining the per site hierarchical scoring scheme? Could you get a better high confidence call set by performing CN calling on an ultra-high depth merge of the 21 replicates (frag size & GC bias variance prevent this?)?

P13 L46 This site scoring approach suffers from the fatal flaw that is it not a metrics of truth, but a metric of concordance with the CN callers chosen. Truth is not determined by orthogonal approach, nor through orthogonal validation, but by consensus. This heavily biases "true positives" towards events/regions that can be detected by multiple callers. For example, a base-pair accurate CN caller would have over 1700 segments (i.e. CN transitions at every breakpoint in the SECQ2 SV truth set) but none of the actually TP <1kbp segments would be classified as TPs by this approach since too many of the callers in this call do not call CN segments this small. Such segments could be identified/validated by the orthogonal ONT data.

P13 L49 This paragraph needs to be expanded to at least a page as there is insufficient detail to evaluate the suitability of the validation approach and definitely not enough to reproduce the results. Details missing include:

- What are 3 orthogonal technologies used? The manuscript in various places mentions ONT, BioNano, Affymetrix microarray & Illumina BeadChip. Which 3 of these 4 were considered validation? [Edit: answered P4 L60]

- What counts as a "calls supported by … orthogonal technologies"? How is this defined for each of the technologies?

- Why were conflicting GAIN/LOSS regions excluded? There's an underlying true CN for every region and you have the orthogonal data to determine which gain/loss/neutral is correct: why exclude?

- How exactly were breakpoints trimmed? What else did you do with breakpoints?

In your P13 L55 example scenario where you go from CN gain directly to CN loss then your orthogonal ONT data will have split reads alignments spanning that break junction. In such scenarios, the actual breakpoint is known and bounds for both the gain and the loss should be adjusted to match the true break junction position. As you haven't included any callers that input SV VCF for use in segmentation, all the CN transition positions are almost guaranteed to be wrong. You already have a high confidence SV call set, why aren't you using that?

P14 L16: Where can I find the actual output artifacts for this manuscript? I can't find any reference to the BED/VCF/xlsx for the CN calls so I am unable to evaluate how good the constructed truth set is.

P17 L13: It's unclear how the Jaccard index is actually calculated. BEDTools jaccard takes BED intervals and overlaps them. There's no classification matching in BEDTools and you have 4(5?) classifications (REF, GAIN, LOSS, (CN-neutral LOH?), NOCALL). How are these matched and how are no-call regions treated? Are taking a bed with the gain and loss regions and calculating the Jaccard index on that? Won't the centromeric calling convention have a huge impact on this? Are you matching a gain with a loss and treating it as a TP? As written, the methods are insufficiently detailed for me to tell whether your Jaccard index is a meaningful metric or not.

FigS4/S5/S9: Are the bands cytobands? Please add an IGV-style triangle/bowtie at the centromeres.

Fig 6A: why are there so many missing data points?

Fig 6B: Does HATCHet really have 0 F-score at intermediate DNA input levels with TruSeq Nano or is this more missing data?

Fig S9: Add an "Excluded/no-call" category so one can determine what your CN high confidence call set is defined over.

Please add an exclusion.bed file wherever you are planning to make your truth bed file available.

Heatmap Figures: is the clustering on the LHS meaningful? If so, do you have any interesting results related to this clustering? Are the gain and loss clusterings similar? What's the deal with the Fig4A ascat/FACETs cluster? Is that something like a ploidy mis-estimation messing up your comparison metrics?

General comments:

Readability is made difficult by critical methods information being split across the methods and results sections and I had to jump between them to understand either section. Please consider copyediting the more methods-focused sections of the results such that the results are readable by themselves without having to refer to the detailed methods.

I'm a strong proponent of reproducible and transparent analysis. This review would have greatly benefited from the presence of a repository (e.g. github) containing the scripts used for generating the data and the analysis script used to generate the table and figures. Please consider replacing the Supp Methods section with a link to such a repository. It's much easier to check your code to see what you've done than to try to infer it from the methods/results sections.

**Reviewer 2**

**Were you able to assess all statistics in the manuscript, including the appropriateness of statistical tests used?**

Yes, and I have assessed the statistics in my report.

**Comments to author:**

In this manuscript titled "Comprehensive Evaluation of Somatic CNV Calling Tools on Benchmark Data Sets" by Masood et al., six CNV callers were evaluated for somatic CNV calling: ascatNgs, CNVkit, FACETS, DRAGEN, HATCHet, and Control-FREEC. They were benchmarked using six NGS short read datasets with various experimental conditions. The study compared reproducibility across sequencing sites, callers, and platforms using 21 WGS replicates. WES datasets were used to compare CNV calls across technologies. A reference CNV call set was established using WGS datasets and validated against three orthogonal technologies: Affymetrix CytoScan, Illumina BeadChip, and Bionano. Additional datasets assessed the effects of non-analytical factors: WGS on library preparation protocols, WGS on tumor sample purity, and FFPE samples at different fixing times.

The authors employed a well-accepted approach in the CNV research community, utilizing consensus results from multiple calling tools to generate a reference call set. The evaluation methods used were rigorous, comprehensive, and appropriate for the study's objectives. The robustness of the findings was demonstrated through the use of three orthogonal validation methods to confirm the reference CNV call set. The benchmarking study extensively explored the impact of various analytical and non-analytical factors on CNV calling accuracy, recall rate, and reproducibility across replicates of WGS and WES runs. These investigations provided valuable insights into the performance and limitations of the somatic CNV callers under different experimental conditions.

The manuscript was well-written and well-structured, facilitating a clear understanding of the research. The bioinformatics and statistical analyses conducted were thorough and comprehensive, aligning well with the study objectives. The figures presented in the study were well-configured, visually intuitive, and effectively conveyed the results.

Additionally, this study generated a high-confidence CNV reference dataset that will serve as a valuable resource for future evaluations of somatic CNV tools. The comprehensive evaluation of six copy number analysis software on WGS and WES, utilizing a large set of sequencing data from multiple sites, significantly enhances our understanding of somatic CNV detection and establishes a benchmark for the research community. The findings and conclusions of this study will make significant contributions to the field of somatic CNV detection, particularly in the context of cancer genomes with notable changes in ploidy.

Questions, suggestions, and areas where clarification is needed

- The samples used in the study, HCC1395 and HCC1395BL, were previously sequenced with long reads (PacBio and Oxford Nanopore) and published in their previous paper (Talsania et al., Genome Biology 2022). However, it is not explicitly stated in the manuscript whether long reads were used in generating the CNV calls. Although the 6 CNV callers are designed for short reads, the inclusion of long reads could assist in validating a reference call set, which could serve as the gold standard in the manuscript.

- It is unclear why these specific six CNV callers were chosen, as well as the criteria used for their selection. The manuscript should provide an explanation for the rationale behind their inclusion. Additionally, a brief description of each of the six callers and the algorithms they employ should be provided to enhance the readers' understanding of the methods utilized.

- The focus of this study was on evaluating somatic CNV calling methods. Previous studies from other research groups have extensively evaluated germline CNV calling, including a study by Gabrielaite et al. (Cancers 2021) that included 50 CNV callers. It is important to introduce the distinction between somatic CNV calling and germline CNV calling and discuss the challenges associated with somatic CNV calling. It would be beneficial to specifically explain in the manuscript how this study helps address these challenges and contributes to the field.

- In a previous study by Coutelier et al. (Eur J Hum Genet 2021), it was recommended to combine four callers, namely Manta, Delly, ERDS, and CNVnator, to enhance CNV detection. What was the reason for excluding all four of these callers from this study?

- The authors stated that the resulting reference call set would be accessible to the public through a dedicated NCBI FTP site. However, I was unable to locate the call set at the specified URL, which is https://ftp-trace.ncbi.nlm.nih.gov/ReferenceSamples/seqc/Somatic_Mutation_WG/.

- All CNV calling results from each caller for all datasets used in this manuscript should be deposited, enabling others to replicate the analysis as needed.

- In addition to the reference CNV call set with genomic locations, it would be beneficial if the authors also provide a list of genes whose CNV status was affected. This would serve as a convenient reference for users to assess the suitability of the reference samples for their own studies.

- It has been stated that this study offers guidance to the cancer research community regarding the selection of sequencing platforms, sample preparation, sequencing coverage, and choice of callers for CNV detection. However, the guidance appears to be embedded within the manuscript, requiring readers to extract it themselves. It would be beneficial to present a clearly laid-out guidance section that provides explicit recommendations.

- The cutoff values for CNV gain and loss calls apparently are not listed for the utilized callers. It is important to include and provide an explanation of the cutoff values in the manuscript.

- Fig. 2B provides a qualitative representation of the Jaccard indices for different groups. It is important to report the dominant Jaccard index numbers for each group in the main text. Additionally, instead of only describing the comparisons between different groups as 'concordance within callers was better than concordance across callers' and 'The distributions of Jaccard indexes within and across sequencing sites were similar,' it is crucial to quantify and report the degree of difference, similarity, or concordance. The statistical significance should also be assessed between the Jaccard index numbers of different groups to provide a comprehensive analysis.

- Although Fig. 3 exists with a legend and the figure itself is present, it was not utilized or mentioned in the main text of the manuscript.

- Fig. 5, which callers achieved relatively better recall and precision should be described in the main text.

- Fig. S7, C and D, what are the y-axis labels, the same as A and B?

- In the Method section, under 'Reproducibility calculation', no justification was provided to exclude certain regions.

- In the Method section, under 'Scoring and categorization of CNV calls', the explanation is unclear, and the inclusion of some equations could help provide further clarification.

**Reviewer 3**

**Were you able to assess all statistics in the manuscript, including the appropriateness of statistical tests used?**

There are no statistics in the manuscript.

**Comments to author:**

This paper conducted comprehensive comparisons between different copy number variation (CNV) callers, CNV platforms/technologies, and other non-analytical factors to benchmark performances of different pipelines in profiling the CNV patterns in terms of detection accuracy, sensitivity, and reproducibility. The results are very interesting as well as important to help the cancer researchers choose the correct pipelines including analytical tools in their studies. However, because lots of concepts and procedures were proposed for diverse comparisons, some parts of the study were not well organized and conveyed in a neat fashion, making the whole manuscript clumsy and disorienting. Further improvements on the intelligibility of the description of the whole work would be very helpful for the readers with little CNV background knowledge to better understand and appreciate the importance of the paper. Below are several of my other comments/concerns.

(1) The study is well designed as described in the first part of the Results. But the corresponding figure (Fig 1) is somehow confusing and vague. I could not obtain clear information from Fig 1 without reading the text about the study design details.

(2) "CNV cluster" seems an important concept in the study, as described in the Method "A CNV cluster is defined as an interval supported by N callsets". However, very fewer results/discussions about CNV cluster were presented.

(3) In the FFPE datasets, it was good that the authors chose samples with different cell fixing time. Did any results show that the optimal cell fixing time from the perspective of the best calling quality? The fixing time is emphasized in the study design, but its relevance to the calling quality was not adequately described in the results and discussion.

(4) In Fig 2, an upset plot of genome region of gain/loss called by each CNV caller will be very helpful (similar to Fig 3C)

(5) An additional table including 6 CNV callers and key parameters used in the study would be very informative.

**Authors Response**

**Point-by-point responses to the reviewers’ comments:**

Reviewer #1:

This manuscript contributed two extremely useful things to the literate: 1) a CNV reference call set for a highly rearranged cancer cell line and 2) an evaluation of popular CNV callers across a range of WGS and WES sequencing parameters.

*Response: We greatly appreciate the acknowledgment of the significance of this study. Following the publication of reference call sets for SNVs and SVs, we have observed a growing interest in CNV call sets for benchmarking purposes. This paper aims to meet this demand by employing a comparable approach to our previous study, establishing a consensus call set, and subsequently validating it using multiple orthogonal methods. Our goal in presenting this CNV reference call set is to offer a valuable resource to the research community, aiding in the advancement and refinement of new tools.*

The key fault in this manuscript is that it fails at 1) as the 'truth' set is just a consensus call set of the callers being compared thus all results are not based on whether a region has a CN change in the cell line has but a measure of the extent to which the CNV callers agree with each other.

*Response: Thank you for bringing attention to this aspect. Due to the limitations inherent in current technologies, there isn't a singular method capable of conclusively identifying regions with CN changes across the entire genome within a cell line without the possibility of false positives or negatives. It's important to clarify that our utilization of a consensus call from six distinct callers served as the initial step to compile likely CNV segments detected via WGS with short-read technology (Illumina). The final establishment of the reference CNV call set was validated and confirmed by three independent orthogonal methods.*

*This methodology mirrors that employed by several prior publications from large consortium studies (GIAB and SEQC2) and is well-regarded within the scientific community. We have previously employed this approach to define reference call sets for SNVs (Fang et al., Nature Biotechnology, 2021, PMID:34504347) and SVs (Talsania et al., Genome Biology, 2022, PMID: 36514120) in the pair of cell lines. The current study adopts a similar methodology to complete the determination of the reference call sets for all variant types, specifically focusing on CNVs as the third variant type in this series.*

CNVs and SVs are fundamentally intertwined semi-orthogonal measurements of the underlying genomic rearrangements. For every SV there needs to be either a compensating SV at the same location or a corresponding copy number transition at that location. Similarly, every (non-telomeric/end of chromosome) CN transition should have an explanatory SV. Understanding the complex genomic rearrangements in this sample (and cancer more generally) involves additional analysis (e.g. JaBba [PMC7912537]/LINX[PMC9903802]) the inputs to which is a consistent SV+CNV call set. That is, one should be able to construct a breakpoint graph.

The sample used here is particularly interesting from this perspective because it's a highly rearranged sample - the authors' consortia has previously published a high confidence SV truth set with 1788 events/breakpoints (c.f. the COLO829 somatic truth set with just 72 high confidence SVs). The authors state "We believe that the high-confident CNV call set on a hyper-diploid genome will be very useful for the cancer research community to benchmark existing tools or develop new algorithms with improved performance." and I completely agree with them. I look forward to having such a truth set on such an interest sample. Unfortunately, this manuscript does not provide such a truth set - there is a >2x discrepancy between the high confidence SV call set (1788 SVs) and this high confidence CNV call set (699 gain/loss/LOH segments).

*Response: We wish to highlight that our SV call set encompasses various types, such as DEL (deletion), DUP (duplication), INS (insertion), INV (inversion), TRA (translocation), and BND (Complex Break Point Events). The SV detection callers primarily sought supporting reads for these events, focusing on higher resolutions that are more sensitive to sub-populations of cells. Previous studies have indicated the highly heterogeneous nature of the cancer cell line HCC1395, characterized by large subclones. Consequently, SV events occurring in smaller cell populations were incorporated into the final SV reference call set, resulting in a larger number of events defined in SV (1788) compared to CNV (699). Notably, the average sizes of DEL (deletion) and DUP (duplication), equivalent to LOSS and GAIN respectively, were considerably smaller (as depicted in Figure R1 and Figure R2).*

*In contrast, CNV callers primarily targeted the LOSS or GAIN of chromosome regions within overall cell populations, factoring in genome ploidy. Therefore, CNV calling exhibited less sensitivity towards events represented by a small fraction of cells or a limited number of supporting reads. Consequently, we assert that the SV and CNV call sets should remain separate entities.*

Figure R1. Segment size distribution of DUP (by SV) and GAIN (by CNV)

Figure R2. Segment size distribution of DEL (by SV) and LOSS (by CNV)

Whilst this discrepancy can be partially explained by the purely depth-based CNV callers compared here being unable to detect small events (thus missing the short CN segments associate with them), this is more of a limitation of the callers chosen than anything fundamental. Callers such as CONSERTING, WEAVER and PURPLE utilise SV breakpoints in their segmentation algorithms and can provide base-pair accurate CN bounds. PURPLE is a particularly notable exclusion as not only is a recent tool used on a very large cohort (PMC9903802/) but the authors used the ploidy estimation from PURPLE in this manuscript but didn't include it in the comparison (at least for the WGS section - PURPLE does not appear to have a WES mode but, as you noted in your discussion, this isn't any different to ascat). This manuscript is completely missing any analysis of the accuracy/resolution of the CN callers, if/how this is improved at high depth (some callers have had parameters changed to reduce resolution at low depth). That is, the smallest segment size each caller can reliably report, and how far off the CN bounds are from the true CN transition/breakpoint position.

*Response: Thank you for highlighting these three CN callers. It's worth noting that the landscape of cancer CNV detection tools has considerably expanded since the publication of the outdated review article (Pirooznia et al., Frontiers in Genetics, 2015). Presently, numerous tools, including recently developed ones like DRAGEN and HATCHet, have emerged.*

*Our selection process involved a comprehensive examination of these tools. We carefully curated six callers chosen for their rigorous evaluation in peer-reviewed publications (Raine et al., Curr Protoc Bioinformatics, 2016; Talevich et al., PLOS Computational Biology 2016; Zaccaria et al., Nature Communications, 2020; Shen et al., Nucleic Acids Research, 2016; Miller et al., Genome Medicine 2015) or their usage in substantial cohort studies (Sakthikumar et al., Genome Biology, 2020; Ma et al., Translational Oncology, 2021; Bailey et al., Nature Communications, 2020; Johansson et al., Nature Communications, 2020; Newell et al., Nature Communications, 2020).*

Furthermore, classifying merely as GAIN/LOSS/LOH is not sufficient for comprehensive somatic genomic rearrangement analysis. Complex events such as a double minutes, breakage-fusion-bridge and chromothripsis are common in many cancers and knowing whether a CN segment is CN 4, 8, or 100 is absolutely essential for differentiating BFB from double minutes.

*Response: Thank you for bringing this to our attention. As suggested, we've included the copy numbers for each gain and loss segment in the reference call set, which can be found in Supplementary Files #2 and #3.*

*To integrate the copy number data, our methodology comprised several steps. Initially, we utilized bedops -partition (https://bedops.readthedocs.io/en/latest/index.html) to fragment all overlapping regions within the 126 WGS call sets (involving 6 callers across 21 replicates) into distinct segments. Subsequently, using bedmap (https://bedops.readthedocs.io/en/latest/content/reference/statistics/bedmap.html), we assigned copy number annotations from these 126 WGS call sets to these discrete segments.*

*Following this assignment process, we computed the median copy number for each individual segment. Then, to streamline the data, we merged smaller concatenated segments that shared the same copy numbers into larger, more coherent segments.*

The results outlined in this manuscript were not provided for review. Notably, I couldn't find the high confidence call set. As such, I am unable to determine whether some of the issues I raise below in the detailed comments are critical issues that materially impact results or are minor issues that can easily be corrected.

In conclusion, this manuscript does not provide the high confidence truth set it purports to, nor does it consider CN caller resolution. I look forward to a revised manuscript that provides the CNV component of the base-pair consistent SV+CNV truth set the cancer genomics bioinformatics research community absolutely needs.

*Response: Thank you for bringing this to our attention. We've successfully uploaded the reference CNV call set and accompanying files generated by the six callers to the designated NCBI ftp site for our working group's use (https://ftp.ncbi.nlm.nih.gov/ReferenceSamples/seqc/Somatic_Mutation_WG/analysis/CNVs/). Moreover, the benchmark sets have been made available on Zenodo at https://doi.org/10.5281/zenodo.10081398. Additionally, the resulting files from the six callers are accessible at https://doi.org/10.5281/zenodo.10081574.*

*As previously mentioned, the SV and CNV call sets represent chromosome alteration events at distinct levels of granularity and cell population, warranting different investigative approaches or algorithms. While the SV reference call set for the cancer cell line has been established and previously published, this manuscript aims to complete the remaining variant type, CNV, specifically for HCC1395. By combining the outcomes of both studies, the research community will have access to reference call sets for benchmarking SV and CNV detection individually.*

Detailed comments:

P2 L29/P9 L14 the estimated ploidy of 2.85 uses the CNV caller PURPLE. Why wasn't GRIDSS2+PURPLE included in this benchmark?

*Response: In our previous study (Zhao et al., Scientific Data, 2021, PMID:34753956), we employed both PURPLE and DRAGEN for ploidy assessment in HCC1395. Remarkably, both callers yielded comparable results. However, in a separate analysis involving high-depth coverage WGS (50X) across 43 B-cell lines derived from lymphoma or multiple myeloma, previously characterized as hyper-diploid or near diploid via cytogenetic analysis (Quentmeier, H. et al., Sci Rep 9, 8218 (2019)), we observed contrasting outcomes. Specifically, as illustrated in Figure R3 and detailed in Table R1, DRAGEN identified all cell lines, barring one, as diploid. In contrast, PURPLE indicated a ploidy level of 3 or higher in 18 out of the 43 cell lines. This discrepancy suggests that PURPLE may tend to overestimate ploidy levels, potentially impacting its accuracy in CNV calling results.*

Figure R3. Ploidy assessment of 43 B cell lines by PURPLE and DRAGEN

Table R1. 43 B cell lines used in ploidy assessment analysis

| **cell line** | **Ploidy (Purple)** | **Ploidy (DRAGEN)** | Cytogenetics |
| --- | --- | --- | --- |
| SU-DHL-8 | 3.84 | 2.21 | hyperdiploid 51(47-53) |
| U-266 | 2.20 | 2.00 | complex hypodiploid - 44(40-46) |
| BALL-1 | 3.78 | 2.05 | complex near-pseudodiploid 46(43-47) |
| MAVER-1 | 2.10 | 1.90 | highly rearranged extreme hypodiploid 40(34-42) |
| BL-70 | 2.80 | 2.00 | hyperdiploid |
| CRO-AP5 | 2.16 | 2.17 | hyperdiploid |
| DOHH-2 | 3.34 | 2.00 | hyperdiploid |
| BC-3 | 2.14 | 2.07 | hyperdiploid 47(43-48) |
| WSU-DLCL2 | 3.44 | 2.01 | hyperdiploid - 47(43-48) |
| U-2973 | 3.64 | 1.99 | hyperdiploid 47(44-48) |
| WSU-FSCCL | 3.53 | 2.06 | hyperdiploid 47(46-50) |
| MHH-PREB-1 | 3.06 | 1.97 | hyperdiploid - 48 |
| CRO-AP6 | 2.16 | 2.09 | hyperdiploid 48(45-48) |
| RI-1 | 2.22 | 2.17 | hyperdiploid - 48-51 |
| U-698-M | 4.30 | 2.14 | hyperdiploid - 49(44-50) |
| GRANTA-452 | 4.08 | 2.17 | hyperdiploid 49(46-50) |
| NU-DHL-1 | 2.46 | 2.12 | hyperdiploid - 51(46-53) |
| OCI-LY1 | 2.14 | 2.05 | hyperdiploid 47(41-48) |
| U-2946 | 3.18 | 2.00 | hyperdiploid -47(43-48) |
| SU-DHL-10 | 2.28 | 2.06 | hyperdiploid- 47(43-48) |
| SC-1 | 3.10 | 2.04 | hyperdiploid- 47(43-48) |
| BL-100 | 3.37 | 2.05 | hyperdiploid 47(44-48) |
| VAL | 3.22 | 2.15 | hyperdiploid- 48(44-50) |
| OCI-LY18 | 2.12 | 2.10 | hyperdiploid 50(48-51) |
| WSU-NHL | 2.52 | 2.02 | hypodiploid - 45(42-45) |
| KMS-12-PE | 2.22 | 1.95 | hypodiploid 42(40-47) |
| NAMALWA | 4.37 | 2.10 | hypodiploid 44(43-46) |
| EHEB | 1.92 | 1.95 | hypodiploid 45(42-46) |
| RAMOS | 3.45 | 2.03 | hypodiploid 45(44-46) |
| DAUDI | 2.19 | 1.95 | near diploid |
| DG-75 | 2.48 | 1.94 | near diploid |
| IM-9 | 2.48 | 2.00 | near diploid - 46(44-46) |
| CARNAVAL | 3.25 | 2.01 | near diploid 46(44-47) |
| CA-46 | 2.69 | 2.08 | near diploid 46(45-48) |
| GUMBUS | 2.65 | 1.91 | near diploid 46(44-46) |
| MN-60 | 3.19 | 1.99 | near diploid 46(45-47) |
| BL-2 | 2.12 | 1.99 | near-diploid 44-47 |
| HT | 2.73 | 1.92 | near-diploid - 46(42-46) |
| MEC-1 | 3.57 | 1.03 | near-diploid - 46(44-47) |
| U-2904 | 2.72 | 2.00 | near-diploid 43-47 |
| L-591 | 2.03 | 2.02 | near-diploid 46(45-48) |
| MOLP-8 | 2.00 | 1.98 | near-diploid karyotype - 40-48 |
| WILL-2 | 2.81 | 2.03 | near-diploid46(43-47) |

P2 L38 How much of this difference is differences in inferred centromeric copy number? No current NGS CNV tools reliably centromeric CN. Some callers don't report centromeric CN and other calls infer a CN across them. Comparing the #bases of non-ref copy number across tools use these difference approaches is results in biased size counts. For a true like-for-like comparison, such regions should be excluded from analysis (and probably the acrocentric short arms as well as there's aren't many 'real' CN calls in these regions either). Does this difference in calling conventions explain the ~300Mb difference between AscatNgs/DRAGEN and CNVKit? I expect that all the analysis is impacted by this issue and the analysis should be repeated excluding these additional known problematic regions.

*Response: Thank you for pointing it out. We should make it more clear in the paper: all gap regions, including centromere and telomere, were excluded in the final call set. We have made a change in the main text and method section. The difference of overall genome CNV regions by each caller thus would not be impact by centromere and telomere regions.*

Fig 2B: what's driving the cross caller differences? Is it AscatNgs vs DRAGEN vs CNVKit clustering at the 0.75 peak? A caller vs caller breakdown would be more informative than just cross vs self. What's driving the inconsistent exons/genes for AscatNgs/DRAGEN/CNVKit? What about for segments? Are the segment differences driven by nested events or actual segmentation differences?

*Response: The inconsistency observed in segment/exon regions across individual callers primarily stemmed from variations in segment calls. Certain callers identified large segments, whereas others detected multiple segments with minor gaps within the same region. This discrepancy arose from differences in the algorithms employed to combine adjacent CN segments and the window sizes utilized in the search for CN regions.*

Fig S2: What are the site abbreviations? "Site" has not been mentioned prior to the point at which this figure is referenced in the main manuscript. It took me a while to realize that red = good and blue=bad - could you invert the colour scheme.

*Response: Thank you for pointing it out.* *We have expanded the abbreviations with their full names in the revised manuscript. We have included an explanation regarding the color scheme for the Jaccard Index in the heatmap for Fig S2, as follows “The color of each cell represents the Jaccard Index, indicating high similarity in red and low similarity in blue. This is calculated based on the length of shared CNV regions divided by the union of CNV regions from two call sets”.*

Fig2C: I don't know what this figure is presenting. There's no colour legend but it goes up to 126 so it looks like it's replicate count but it could be a cumulative segment count. The X axis is also label "Replicate" so it's really unclear what's being presented. All I can get from this is that ascatNgs/DRAGEN are the most consistent then CNVkit then FACETS and with Control-FREEC/HATCHet not great. What does "CNV Regions" mean and what's the colour actual represent? Please provide more clarity on this figure.

*Response: Thank you for your suggestion. This figure illustrates the consistency of CNV regions detected across 126 replicates from 7 sites and 6 callers. We have moved the description of this figure to the section entitled “Concordance of CNV Calls across WGS Replicates” in the revised manuscript. Additionally, we have updated the figure legend to provide a clearer and comprehensive explanation*.

P4 L25: please provide a sentence or two in the main body about the meaning of your site score. I can't tell at this point whether a site score of 3 good or bad. See also my P13 L17 comment about the multiple meanings of "site" in this manuscript.

*Response: Thank you for pointing it out. The clarification regarding the significance of site scores and the methodology for obtaining them has been included in the revised manuscript.*

P5: See P13 comments regarding insufficient detail on how validation was performed and what was needed for a segment to be considered 'validated'. The high confidence call set would benefit considerably from validation of the CN transition locations with the long read data you already have.

*Response: Thank you for pointing it out. We have made changes in the text and provided more detail on how validation was performed in Supplementary Figure S4C.*

P5 L17: The number of calls only makes sense in the context of consistency with your high confidence SV call set. How consistent are your CN transitions with your previously published SV call set? Note that this consistency can be used as a proxy for the joint quality of SV/CNV call sets (e.g. PMID: 3425323 Fig 3).

*Response: Please refer to our response to your comments on SV vs CNV call sets.*

Once you include actual estimated CNs, you can further evaluate the consistency of the CNs. For example, if there's a CN transition from 3 to 1 at a position with an explanatory breakpoint, the other side of that breakpoint should also have a copy number delta of 2.

*Response: Yes, as you pointed out and shown in an IGV view of chr13q in Figure R4, clear boundaries and breakpoints were observed between segments with different copy number calls.*

Figure R4. Copy number of CNV gain regions on Chr13q. IGV display of normalized reads coverage and B-allele frequency (BAF) of chromosome 13q arm of randomly selected four WGS runs (NV_3, EA_1, NC_1, NS_2)

P5 L23 how is this validated? Your methods state you use bedtools multiinter to break up intervals but that's going to result in heaps of tiny clusters/segments at the start/end of each actual CN segment. Figure S10B indicates that you might perform some sort of merging, but it's not mentioned anywhere in the manuscript, and the unreferenced Fig S10B could also be an undocumented explanation of your Jaccard index calculation.

*Response: Thank you for pointing it out. We included the validation details in the revised manuscript, and we have also made updates to Fig S10 (now Fig S4C) along with its corresponding figure legend.*

P5 L37 How much of the WGS/WES difference is driven by calling convention? Did you compare the genome-wide inferred CN from WES with WGS, or have you limited your WES vs WGS analysis to only the WES targeted regions? Generally speaking, the off-target WES CN isn't anywhere near as important as getting the exonic CN correct. What's the Jaccard index between WES and WGS in just the on-target regions?

*Response: We limited our WES vs WGS analysis to only the WES targeted regions and the Jaccard index between WES and WGS was based on WES targeted regions.*

P5 L52: what's driving this? Ploidy mis-estimation?

*Response: Thank you for pointing this out. To investigate this, we computed overall ploidy of each run as the length-weighted average of total copy number across the full genome. Both Control-FREEC and Hatchet show part of the samples with ploidy significantly different from 2.85.*

| Sample | platform | Computed ploidy | |
| --- | --- | --- | --- |
|  |  | HATChet | Control-FREEC |
| WES_EA_1 | WES | 3.01 | 4.26 |
| WES_FD_1 | WES | 3.09 | 4.07 |
| WES_FD_2 | WES | 3.29 | 3.49 |
| WES_FD_3 | WES | 2.80 | 3.67 |
| WES_IL_1 | WES | 3.32 | 3.53 |
| WES_IL_2 | WES | 2.72 | 4.14 |
| WES_IL_3 | WES | 2.57 | 4.25 |
| WES_LL_1 | WES | 2.82 | 3.50 |
| WES_NC_1 | WES | 3.63 | 4.70 |
| WES_NV_1 | WES | 3.13 | 4.05 |
| WES_NV_2 | WES | 3.06 | 4.05 |
| WES_NV_3 | WES | 3.10 | 4.30 |
| WGS_EA_1 | WGS | 2.94 | 4.99 |
| WGS_FD_1 | WGS | 2.95 | 3.09 |
| WGS_FD_2 | WGS | 3.00 | 5.11 |
| WGS_FD_3 | WGS | 2.88 | 5.10 |
| WGS_IL_1 | WGS | 2.85 | 3.07 |
| WGS_IL_2 | WGS | 2.88 | 3.24 |
| WGS_IL_3 | WGS | 2.97 | 3.09 |
| WGS_LL_1 | WGS | 2.93 | 5.05 |
| WGS_NC_1 | WGS | 3.10 | 3.10 |
| WGS_NS_1 | WGS | 3.14 | 3.09 |
| WGS_NS_2 | WGS | 2.43 | 4.96 |
| WGS_NS_3 | WGS | 3.19 | 2.98 |
| WGS_NS_4 | WGS | 1.48 | 3.15 |
| WGS_NS_5 | WGS | 3.13 | 4.79 |
| WGS_NS_6 | WGS | 3.00 | 2.96 |
| WGS_NS_7 | WGS | 3.07 | 2.87 |
| WGS_NS_8 | WGS | 1.49 | 5.02 |
| WGS_NS_9 | WGS | 3.04 | 3.02 |
| WGS_NV_1 | WGS | 2.82 | 3.13 |
| WGS_NV_2 | WGS | 2.82 | 3.40 |
| WGS_NV_3 | WGS | 2.83 | 3.14 |

Table R2. Computed ploidy results from HATChet and Control-FREEC in WES and WGS data set

*Given that ploidy has a non-negligible effect on CNV calling, we think the most plausible root cause for this variability is the mis-estimation of ploidy.*

P5 L37: What is the gain concordance stratified by CN? Is the higher concordance of gains vs loss caused by some regions being highly amplified thus being really easy to call as gains? Are the CN=3 as good as CN=1? CN=4 the same as CN=0?

*Response: Thank you for the suggestion. With inclusion of copy # in the reference CNV call set, we tried to extrapolate the calling consistency. However, as shown in Figure R5, copy numbers detected by ascatGNS, CNVkit, and DRAGEN show consistency among replicates, while Control-FREEC, FACETS, and HATCHet have larger variation. Notably, we observed that copy numbers detected by CNVkit and FACETs are higher compared to other callers. Thus, it is reasonable to believe that less concordance would be expected when stratified by CN as regions with same copy number calls by all six callers would be a small fraction of the concordant gain segment.*

Figure R5. Distribution of genomic regions characterized with different copy numbers by six callers

P6 L15: Does fixing time matter? Fig5 doesn't label the FFPE fixing time so I can't tell if there's a trend.

*Response: We've extrapolated the calling consistency based on FFPE fixing time, illustrated in Supplementary Figure S12. Our analysis indicates that an increase in FFPE fixing time does not notably impact the accuracy of CNV calls. Moreover, when comparing WGS and WES datasets derived from FFPE samples, we observe a higher accuracy in CNV calls within the WGS datasets. Notably, the accuracy is heavily reliant on the choice of caller utilized. For example, CNVkit displayed superior performance in detecting copy number gains, while HATCHet and Control-FREEC exhibited comparatively lower performance levels.*

Figure R6. The F1 score for copy number gain (A) and copy number loss (B) under varying FFPE fixing time.

P6 L20: are you excluding regions that the CNV caller makes no call in your prec/recall calculations? NB: not all callers make a REF vs no-call distinction, but I know at least DRAGEN does if you give it the appropriate parameters.

*Response: All considered callers in this analysis report REF calls. We excluded gap regions in the precision/recall calculations.*

P6 L35: How did you make >100x coverage data sets? From what I can tell, all replicates are less than 100x depth. Which samples and what merging logic did you use? Not really an issue for these callers, but if you add a SV+CNV pipeline such as GRIDSS2/PURPLE into the comparison, then merging sample with dissimilar fragment size distributions could be problematic on the SV calling side of things (I think you have enough similar replicates that this won't be an issue in practice).

*Response: By combining multiple replicates runs (merging three bam files), we generated a WGS run with more than 100X coverage. We have modified the text and made it clear in the main text. All replicates were using same library preparation protocol and thus in similar fragment size distributions (Xiao et al., Nature Biotechnology, 2021 Sep;39(9), Suppl_Data_File_#1).*

P6 L38 could this performance plateau at 30x be converted into equal performance with a narrow window size thus an increased ability to detect small CN segments?

*Response: In theory, it could identify a set of CN with smaller segments. However, it cannot provide as evidence of "real" CN and simply reflect as artifacts due to insufficient reads coverage.*

P6 L52: So are these variable HATCHet results intrinsic caller instability that is actually independent of variable under test? How similar are the HATCHet results if you take your 90x coverage replicates and randomly split the reads into three 30x non-overlapping subsets and run HATCHet? If there's variance in these three runs then it's likely that the instability is inherit in the HATCHet algorithm.

*Response: We observed less-ideal performance of HATCHet on our data sets and don't have good explanation what cause it. Our observation showed that HATCHet did not make ploidy assessment correctly in those data set with inconsistent results. We wish to have better insight of its bad performance; however, complete dissecting HATCHet is not the focus of this manuscript and we are not sure if our data sets are suitable for this purpose.*

P7 L38. Please expand on ploidy estimate in the results section. Burying this important finding halfway through the discussion does disservice to just how important ploidy estimation is in somatic CNV calling. Ploidy estimation is a hard problem and even non-fold-change based approaches suffer from choosing purity/ploidy that half/double of the actual value.

*Response: Thank you for stress the value of this discovery! We expanded this section and moved up to the result section “Concordance of CNV calls across WGS replicates”.*

P7 L57 With an actual ploidy results section, more of the ploidy discussion can be dedicated addressing various ploidy-based limitation and their practical impacts on real pipelines. E.g. do real-world CNVKit-based CN pipelines need to use PURPLE (or an equivalent tool) for purity/ploidy estimation like you have done here? Are ploidy estimates less reliable for WES vs WGS? (some of these questions request an entire cohort to comprehensively address).

*Response: By default, CNVkit doesn’t require ploidy-awareness. As shown in Figure R3 above, PURPLE tends to over-estimate the ploidy of cancer genome. Also, as shown in Table R2, demonstrated by Control-FREEC, ploidy estimates in WES were much less reliable than in WGS.*

P9 Not really needed in this manuscript but it would be useful to users if tool authors could update their documentation/default settings to reflect any non-standard best-practice parameters used in this comparison.

*Response: All key parameters are summarized in Table S4.*

P13 L17. It is here that I find site means the sequencing centre performing the sequencing. Until this point I thought it was some classification of genomic site (e.g. STR, VNTR) and I just didn't know what the abbreviations were. Using something like "Sequencing Center" (like you do at P4 L32) would have been less confusing for me. Also, I still don't know what FD IL NV NS EA NV & LL actually mean.

*Response: Thank you for pointing it out. We have made changes in the text in the “Study design” section. Here is abbreviation of sequencing sites: FD- Fudan University, IL- Illumina-HiSeq, NV- Novartis, NS- Illumina-NovaSeq, EA-EATRIS, NC- National Cancer Institute, LL- Loma Linda University.*

P13 L27 "reprehensibility". Did you mean reproducibility?

*Response: Thank you for pointing it out. We have fixed this typo.*

P13 L30 I still don't understand how site score is calculated. Why split into 5 groups? Why are the groups named SUPP #1 to SUPP #5? Why did group 4 have 9 NS replicates? How does the method handle missing data (regions not called with any CN by a particular caller)? (Edit: I found meaningful group labels in Fig S10).

*Response: Thank you for your suggestions. We have updated the section entitled “Partitioning, scoring, and assigning confidence levels for CNV intervals”. The site score is calculated as follow:*

*“The confidence levels for each CNV interval were assessed based on reproducibility at three levels: within lab, cross lab, and cross callers. A site score is assigned based on reproducibility within the lab and cross lab. Following that, confidence levels are assigned based on the reproducibility across callers. Six site scores were initially assigned, considering reproducibility across 21 replicates from 7 sites for each of the six callers. To obtain a site score, the 21 replicates were divided into five groups for each caller, as illustrated in Figure S4A. Replicates from the same site are expected to yield consistent results. We first assessed reproducibility among replicates within the same site. Consequently, three replicates from FD were grouped as Group 1, three replicates from IL as Group 2, three replicates from NV as Group 3, nine replicates from NS as Group 4, and one replicate each from EA, LL and NC were designed as Group 5. A CNV interval received an intermediate score based on the number of supported replicates within each group, ranging from 0 to 3. For example, in a group with three replicates from a specific caller, is CNVs are called by call set #1 and #2 but not #3, the intermediate score before being added up as a site score is 2. In Group 4, consisting of nine replicates from NS, their intermediate score was divided by 3. The site scores for each caller were then aggregated across the five groups, and the cumulative site score was converted to a range of 0 to 3: 10-15 to 3, 7-9 to 2, 4-6 to 1, 0-3 to 0. A lower site score indicated less concordance among replicates and sequencing sites (Fig S5A).”*

*We revised “SUPP #1 to SUPP #5 to “Group Score #1 to #5” in supplementary Figure S5A for easier understanding. This figure shows the combination of site scores instead of a specific score for a specific group. The reproducibility is assessed on three levels: within lab, cross lab, and cross callers. Nine replicates were generated in NS, and the same CNV calls are expected to be detected from these replicates. Therefore, we grouped them all in the same group, labeled as Group #4. For a CNV interval, the intermediate score is initially assigned based on the number of supported replicates within each group. For instance, in a group with three replicates from a specific caller, if CNVs are called by call set #1 and #2 but not #3, the intermediate score before being added up as a site score is 2.*

Given that your results indicate that sequencing centre doesn't really matter for the results, what's the value in retaining the per site hierarchical scoring scheme? Could you get a better high confidence call set by performing CN calling on an ultra-high depth merge of the 21 replicates (frag size & GC bias variance prevent this?)?

*Response: Thank you for your insightful comments and suggestions. In our recent study (Zheng et al., Nature Biotechnology, 2023, PMID:37679543), we meticulously identified variations in wet-lab proficiency during omics data generation. Notably, the sequencing centers consistently delivered high-quality sequencing datasets (Zhao et al., Scientific Data, 2021, PMID:34753956), diminishing the impact of specific sequencing centers on our results. As previously mentioned, our assessment of reproducibility encompasses three levels of analysis, ranging from less to more stringent: replicates within the same sequencing center, cross-lab evaluations employing the same caller, and cross-caller comparisons. As depicted in Figure 5, we observed that the accuracy of CNV calls remained unaffected by varying sequencing depths, whether lower or higher.*

P13 L46 This site scoring approach suffers from the fatal flaw that is it not a metrics of truth, but a metric of concordance with the CN callers chosen. Truth is not determined by orthogonal approach, nor through orthogonal validation, but by consensus. This heavily biases "true positives" towards events/regions that can be detected by multiple callers. For example, a base-pair accurate CN caller would have over 1700 segments (i.e. CN transitions at every breakpoint in the SECQ2 SV truth set) but none of the actually TP <1kbp segments would be classified as TPs by this approach since too many of the callers in this call do not call CN segments this small. Such segments could be identified/validated by the orthogonal ONT data.

*Response: Thank you for your valuable comments and suggestions. To construct the CNV reference call set, we initially categorized CNV regions into four confidence levels (strong, medium, weak, and neutral) based on the reproducibility of 126 WGS call sets. Subsequently, the CNV call set underwent determination through orthogonal technologies such as Affymetrix CytoScan, Illumina BeadChip, and Bonino, outlined in the Results section titled “Validation of consensus CNV calls with orthogonal technologies.”*

*In our study, the CNV reference call set comprises strong-evidence calls alongside medium and weak-evidence calls supported by at least two of the three orthogonal technologies. However, calls categorized as neutral evidence were excluded from the high-confidence call sets. Notably, as only one replicate was generated by Affymetrix and Bonino, relying solely on these individual technologies to support CNV calls isn’t substantiated enough to consider them as definitive truth sets. Consequently, our CNV reference call set might overlook genuine CNV calls uniquely identified by a specific caller or technology.*

*We acknowledge the evolving landscape of technologies and bioinformatic algorithms. Therefore, we intend to update the CNV reference call set to progressively approach a more comprehensive somatic CNV profile of the HCC1395 genome. Furthermore, as depicted in Figure 4 and Figure S8, the CNVs detected by Bonino closely align with the strong-evidence calls identified through NGS. The chromosome plot also indicates a consistency where no long segments detected by Bonino were missed by NGS.*

P13 L49 This paragraph needs to be expanded to at least a page as there is insufficient detail to evaluate the suitability of the validation approach and definitely not enough to reproduce the results. Details missing include:

- What are 3 orthogonal technologies used? The manuscript in various places mentions ONT, BioNano, Affymetrix microarray & Illumina BeadChip. Which 3 of these 4 were considered validation? [Edit: answered P4 L60]

*Response: Thank you for your comments and suggestions. We moved the validation details to the Results section entitled “Validation of Consensus CNV Calls with Orthogonal Technologies” and revised the “Partitioning, Scoring, and Assigning Confidence Levels for CNV Intervals”. Three orthogonal technologies have been used.*

- What counts as a "calls supported by … orthogonal technologies"? How is this defined for each of the technologies?

*Response: Thank you for pointing it out. The validation of a CNV region was considered successful if the same region was identified by any of the orthogonal technologies, as illustrated in updated Figure S4C.*

- Why were conflicting GAIN/LOSS regions excluded? There's an underlying true CN for every region and you have the orthogonal data to determine which gain/loss/neutral is correct: why exclude?

*Response: Thank you for highlighting this aspect. To reconcile conflicting CNV regions where both gains and losses were simultaneously identified, we compared CNV gain and loss calls from the NGS consensus catalog with those derived from orthogonal technologies. For instance, when a region identified as a strong-evidence NGS CNV gain was designated as a loss region by orthogonal technologies without any supportive evidence as a gain region, these conflicting NGS CNV regions were omitted from the benchmark set (Examples #2 and #4 in Figure R7).*

*Conversely, if a strong-evidence NGS CNV gain region exhibited gain calls supported by orthogonal technologies, even if some orthogonal technologies indicated loss calls, the strong-evidence NGS CNV gain region would be retained in the benchmark set (Examples #1 and #3 in Figure R7).*

Figure R7. Examples of conflicting regions.

- How exactly were breakpoints trimmed? What else did you do with breakpoints?

*Response: Thank you for pointing it out. Breakpoints were trimmed based on evidence from orthogonal technologies. For instance, if a gain end and a loss end overlapped with each other, and the gain end was supported by any orthogonal technologies, the gain end would be retained, while the loss end would be trimmed, as illustrated in Figure R8.*

Figure R8. Schematic plot for breakpoint trimming based on orthogonal technologies.

In your P13 L55 example scenario where you go from CN gain directly to CN loss then your orthogonal ONT data will have split reads alignments spanning that break junction. In such scenarios, the actual breakpoint is known and bounds for both the gain and the loss should be adjusted to match the true break junction position. As you haven't included any callers that input SV VCF for use in segmentation, all the CN transition positions are almost guaranteed to be wrong. You already have a high confidence SV call set, why aren't you using that?

*Response: As stated in previous response, SV and CNV call set represent chromosome alteration events at different granularity level and cell population level and thus should be investigated with different approaches or algorithms. We examined the relationship between AMP/DEL and GAIN/LOSS and found that most AMP/DEL events may not share the same breakpoints with GAIN/LOSS (Figure R9).*

Figure R9. Chromosome plot of SV AMP benchmark set and CNV gain benchmark set.

P14 L16: Where can I find the actual output artifacts for this manuscript? I can't find any reference to the BED/VCF/xlsx for the CN calls so I am unable to evaluate how good the constructed truth set is.

*Response: Thank you for pointing it out. We have uploaded all resulting files from six callers onto the NCBI ftp site (https://ftp.ncbi.nlm.nih.gov/ReferenceSamples/seqc/Somatic_Mutation_WG/analysis/CNVs/). The resulting files from the six callers are also available at* [*https://doi.org/10.5281/zenodo.10081574*](https://doi.org/10.5281/zenodo.10081574)*.*

P17 L13: It's unclear how the Jaccard index is actually calculated. BEDTools jaccard takes BED intervals and overlaps them. There's no classification matching in BEDTools and you have 4(5?) classifications (REF, GAIN, LOSS, (CN-neutral LOH?), NOCALL). How are these matched and how are no-call regions treated? Are taking a bed with the gain and loss regions and calculating the Jaccard index on that? Won't the centromeric calling convention have a huge impact on this? Are you matching a gain with a loss and treating it as a TP? As written, the methods are insufficiently detailed for me to tell whether your Jaccard index is a meaningful metric or not.

*Response: Thank you for your comments and suggestions. We have updated the description of how the Jaccard index is calculated as follows:*

*“There are three types of variants in the original CNV call set, including gain calls, loss calls and LOH calls. We divided the original CNV call set into gain calls, loss calls and loh calls, then computed their reproducibility separately. The Jaccard index was utilized to evaluate the reproducibility or similarity of two call sets. It is calculated as the intersection of the length of genomic regions of CNV calls between two call sets divided by the union length. The Jaccard index was computed using the “jaccard” command of BedTools. When assessing the reproducibility of WGS call sets at the segment level, all CNV segments on the whole genome were included. CNVs on the p-arm of chr6, q-arm of chr16, chrX and gap regions (including centromere regions and telomere regions) were excluded from this analysis. In evaluations at the gene and exon levels, CNV-affected regions reproducibility within annotated gene or exon regions by Refseq were retained. For comparisons of reproducibility between WGS and WES datasets, CNV-affected regions within exons annotated by Refseq were considered.”*

FigS4/S5/S9: Are the bands cytobands? Please add an IGV-style triangle/bowtie at the centromeres.

*Response: The bands represent cytobands, and the figures were generated using the R package “karyoploteR”, illustrating whole-genome representations with CNV regions plotted above them. The read portion in the middle of bands signifies the centromeres.*

Fig 6A: why are there so many missing data points?

*Response: No CN regions has been identified in these samples.*

Fig 6B: Does HATCHet really have 0 F-score at intermediate DNA input levels with TruSeq Nano or is this more missing data?

*Response: Again, no CN regions has been identified by HATCHet in these samples.*

Fig S9: Add an "Excluded/no-call" category so one can determine what your CN high confidence call set is defined over.

*Response: Thank you for pointing it out. We have added one track for “Excluded/no-call" category.*

Please add an exclusion.bed file wherever you are planning to make your truth bed file available.

*Response: Thank you for pointing it out. The bed file has been uploaded to NCBI ftp site as well.*

Heatmap Figures: is the clustering on the LHS meaningful? If so, do you have any interesting results related to this clustering? Are the gain and loss clusterings similar? What's the deal with the Fig4A ascat/FACETs cluster? Is that something like a ploidy mis-estimation messing up your comparison metrics?

*Response: ascat/FACET showed high similarity whereas a subset of HATCHet and Contro-FREEC showed distinct cluster, coincidentally with wrongly ploidy assessment.*

General comments:

Readability is made difficult by critical methods information being split across the methods and results sections and I had to jump between them to understand either section. Please consider copyediting the more methods-focused sections of the results such that the results are readable by themselves without having to refer to the detailed methods.

*Response: Thank you for the suggestions. We have moved some paragraphs/descriptions to the Methods section.*

I'm a strong proponent of reproducible and transparent analysis. This review would have greatly benefited from the presence of a repository (e.g. github) containing the scripts used for generating the data and the analysis script used to generate the table and figures. Please consider replacing the Supp Methods section with a link to such a repository. It's much easier to check your code to see what you've done than to try to infer it from the methods/results sections.

*Response: Thank you for pointing it out. We have uploaded all related resulting files, reference call sets, and scripts to generate figures and tables to NCBI ftp site (*[*https://ftp.ncbi.nlm.nih.gov/ReferenceSamples/seqc/Somatic_Mutation_WG/analysis/CNVs/*](https://ftp.ncbi.nlm.nih.gov/ReferenceSamples/seqc/Somatic_Mutation_WG/analysis/CNVs/)*) and zenodo (https://zenodo.org/records/10198327) that is accessible by public.*

Reviewer #2: In this manuscript titled "Comprehensive Evaluation of Somatic CNV Calling Tools on Benchmark Data Sets" by Masood et al., six CNV callers were evaluated for somatic CNV calling: ascatNgs, CNVkit, FACETS, DRAGEN, HATCHet, and Control-FREEC. They were benchmarked using six NGS short read datasets with various experimental conditions. The study compared reproducibility across sequencing sites, callers, and platforms using 21 WGS replicates. WES datasets were used to compare CNV calls across technologies. A reference CNV call set was established using WGS datasets and validated against three orthogonal technologies: Affymetrix CytoScan, Illumina BeadChip, and Bionano. Additional datasets assessed the effects of non-analytical factors: WGS on library preparation protocols, WGS on tumor sample purity, and FFPE samples at different fixing times.

The authors employed a well-accepted approach in the CNV research community, utilizing consensus results from multiple calling tools to generate a reference call set. The evaluation methods used were rigorous, comprehensive, and appropriate for the study's objectives. The robustness of the findings was demonstrated through the use of three orthogonal validation methods to confirm the reference CNV call set. The benchmarking study extensively explored the impact of various analytical and non-analytical factors on CNV calling accuracy, recall rate, and reproducibility across replicates of WGS and WES runs. These investigations provided valuable insights into the performance and limitations of the somatic CNV callers under different experimental conditions.

The manuscript was well-written and well-structured, facilitating a clear understanding of the research. The bioinformatics and statistical analyses conducted were thorough and comprehensive, aligning well with the study objectives. The figures presented in the study were well-configured, visually intuitive, and effectively conveyed the results.

Additionally, this study generated a high-confidence CNV reference dataset that will serve as a valuable resource for future evaluations of somatic CNV tools. The comprehensive evaluation of six copy number analysis software on WGS and WES, utilizing a large set of sequencing data from multiple sites, significantly enhances our understanding of somatic CNV detection and establishes a benchmark for the research community. The findings and conclusions of this study will make significant contributions to the field of somatic CNV detection, particularly in the context of cancer genomes with notable changes in ploidy.

*Response: Thank you very much for your insightful comments. We greatly appreciate the acknowledgment of the significance of this study. Following the publication of reference call sets for SNVs and SVs, we have observed a growing interest in CNV call sets for benchmarking purposes. This paper aims to meet this demand by employing a comparable approach to our previous study, establishing a consensus call set, and subsequently validating it using multiple orthogonal methods. Our goal in presenting this CNV reference call set is to offer a valuable resource to the research community, aiding in the advancement and refinement of new tools.*

Questions, suggestions, and areas where clarification is needed

-	The samples used in the study, HCC1395 and HCC1395BL, were previously sequenced with long reads (PacBio and Oxford Nanopore) and published in their previous paper (Talsania et al., Genome Biology 2022). However, it is not explicitly stated in the manuscript whether long reads were used in generating the CNV calls. Although the 6 CNV callers are designed for short reads, the inclusion of long reads could assist in validating a reference call set, which could serve as the gold standard in the manuscript.

*Response:*

*Thank you for highlighting this aspect. While we did obtain long reads from PacBio and Oxford Nanopore for both cell lines, the coverage across the entire genome was relatively low, less than 10-fold. In our previous paper (Talsania et al., Genome Biology 2022), we established an SV reference call set covering various types including DEL (deletion), DUP (duplication), INS (insertion), INV (inversion), TRA (translocation), and BND (Complex Break Point Events). The SV detection primarily relied on identifying supporting reads for these events, focusing on higher resolutions that are more sensitive to sub-populations of cells.*

*The cancer cell line HCC1395, as noted in previous studies (Fang et al., Nature Biotechnology 2021), is highly heterogeneous, characterized by large subclones. Consequently, SV events occurring in smaller* *cell populations were integrated into the final SV reference call set, resulting in a higher count of events defined in SV (1788) compared to CNV (699). Notably, the average sizes of DEL (deletion) and DUP (duplication), equivalent to LOSS and GAIN respectively, were notably smaller, as depicted in Figure R1 and Figure R2.*

*In contrast, CNV callers primarily focused on detecting gain or loss in chromosome regions across overall cell populations, considering genome ploidy. As a result, CNV calling exhibited less sensitivity towards events represented by a small fraction of cells or a limited number of supporting reads. Due to the low coverage of long reads, we determined they might not be sufficient for accurate CNV calling.*

Figure R1. Segment size distribution of DUP (by SV) and GAIN (by CNV)

Figure R2. Segment size distribution of DEL (by SV) and LOSS (by CNV)

-	It is unclear why these specific six CNV callers were chosen, as well as the criteria used for their selection. The manuscript should provide an explanation for the rationale behind their inclusion. Additionally, a brief description of each of the six callers and the algorithms they employ should be provided to enhance the readers' understanding of the methods utilized.

*Response:*

*Thank you for suggestion. It's worth noting that the landscape of cancer CNV detection tools has considerably expanded since the publication of the outdated review article (Pirooznia et al., Frontiers in Genetics, 2015). Presently, numerous tools, including recently developed ones like DRAGEN and HATCHet, have emerged.*

*Our selection process involved a comprehensive examination of these tools. We carefully curated six callers chosen for their rigorous evaluation in peer-reviewed publications (Raine et al., Curr Protoc Bioinformatics, 2016; Talevich et al., PLOS Computational Biology 2016; Zaccaria et al., Nature Communications, 2020; Shen et al., Nucleic Acids Research, 2016; Miller et al., Genome Medicine 2015) or their usage in substantial cohort studies (Sakthikumar et al., Genome Biology, 2020; Ma et al., Translational Oncology, 2021; Bailey et al., Nature Communications, 2020; Johansson et al., Nature Communications, 2020; Newell et al., Nature Communications, 2020).*

*We have incorporated above justification in the manuscript.*

-	The focus of this study was on evaluating somatic CNV calling methods. Previous studies from other research groups have extensively evaluated germline CNV calling, including a study by Gabrielaite et al. (Cancers 2021) that included 50 CNV callers. It is important to introduce the distinction between somatic CNV calling and germline CNV calling and discuss the challenges associated with somatic CNV calling. It would be beneficial to specifically explain in the manuscript how this study helps address these challenges and contributes to the field.

*Response: Will emphasize in the main text that this manuscript is about "somatic CNV" with match normal as the genome background.*

-	In a previous study by Coutelier et al. (Eur J Hum Genet 2021), it was recommended to combine four callers, namely Manta, Delly, ERDS, and CNVnator, to enhance CNV detection. What was the reason for excluding all four of these callers from this study?

*Response:*

*Thank you for the suggestion. In the study by Courtelier et al., the authors employed a combination of four callers to enhance CNV germline variant calling. It's important to note that the conclusions and recommendations drawn from this study might not directly translate to somatic CNV detection in cancer cells. Specifically, all four callers used in their research were intended for germline CNV variant calling and therefore were not included in our study.*

-	The authors stated that the resulting reference call set would be accessible to the public through a dedicated NCBI FTP site. However, I was unable to locate the call set at the specified URL, which is <https://ftp-trace.ncbi.nlm.nih.gov/ReferenceSamples/seqc/Somatic_Mutation_WG/>.

*Response: Thank you for pointing it out. We have uploaded all related resulting files, reference call sets, and scripts to generate figures and tables to NCBI ftp site (https://ftp.ncbi.nlm.nih.gov/ReferenceSamples/seqc/Somatic_Mutation_WG/analysis/CNVs/) that is accessible by public, and also Zenodo at https://doi.org/10.5281/zenodo.10081398.*

-	All CNV calling results from each caller for all datasets used in this manuscript should be deposited, enabling others to replicate the analysis as needed.

*Response: Thank you for pointing it out. We have uploaded all related resulting files, reference call sets, and scripts to generate figures and tables to NCBI ftp site (https://ftp.ncbi.nlm.nih.gov/ReferenceSamples/seqc/Somatic_Mutation_WG/analysis/CNVs/) that is accessible by public, Zenodo at https://doi.org/10.5281/zenodo.10081574.*

-	In addition to the reference CNV call set with genomic locations, it would be beneficial if the authors also provide a list of genes whose CNV status was affected. This would serve as a convenient reference for users to assess the suitability of the reference samples for their own studies.

*Response: Thank you for pointing it out. We have looked up genes in the regions of reference CNV call set and included in the paper as supplementary file #1. Overall, 15,189 of gene are in the regions with GAIN call, 441 of genes in the regions with LOSS call, and 14,760 of gene in the regions with LOH call.*

-	It has been stated that this study offers guidance to the cancer research community regarding the selection of sequencing platforms, sample preparation, sequencing coverage, and choice of callers for CNV detection. However, the guidance appears to be embedded within the manuscript, requiring readers to extract it themselves. It would be beneficial to present a clearly laid-out guidance section that provides explicit recommendations.

*Response: Thank you for the suggestions. In the original study design, each sequencing center preform WGS and WES runs with protocol recommended by the manufacture recommended for library preparation and sequencing. However, each sequencing center had their own preference of DNA fragmentation, library QC and quantification, sample loading and PCR amplification. As a result, library processing was not well-controlled. Therefore, we are not in the position to provide in detail on how wet-lab process should be done. Therefore, we revised our conclusion with following statement.*

*“With performance matrices on various experimental conditions, this study raises awareness within the cancer research community for the selection of sequencing platforms, sample preparation, sequencing coverage, and the choice of CNV detection tools.”*

-	Library preparation method does not show noticeable impact on this high purity cell line.

-	The cutoff values for CNV gain and loss calls apparently are not listed for the utilized callers. It is important to include and provide an explanation of the cutoff values in the manuscript.

*Response: Thank you for your comments and suggestions. We have added the description of copy number cutoff values.*

-	Fig. 2B provides a qualitative representation of the Jaccard indices for different groups. It is important to report the dominant Jaccard index numbers for each group in the main text. Additionally, instead of only describing the comparisons between different groups as 'concordance within callers was better than concordance across callers' and 'The distributions of Jaccard indexes within and across sequencing sites were similar,' it is crucial to quantify and report the degree of difference, similarity, or concordance. The statistical significance should also be assessed between the Jaccard index numbers of different groups to provide a comprehensive analysis.

*Response: Thank you very much for your comments and suggestion. We have updated the qualitative representation of the Jaccard index.*

-	Although Fig. 3 exists with a legend and the figure itself is present, it was not utilized or mentioned in the main text of the manuscript.

*Response: Thank you for your comments and suggestions. We had added the description of previous Fig 3 (Fig 4 in the revised manuscript) as follows:*

*“For CNV gain and LOH, all three orthogonal technologies well supported the NGS strong evidence regions (Fig 4 and Fig S7). For CNV losses, Illumina BeadChip and BioNano supported the NGS strong evidence regions, while Affymetrix CytoScan detected more regions consistent with NGS neutral evidence regions (Fig S8). However, as only one replicate was generated by Affymetrix CytoScan, the evidence of CNV calls uniquely by this technology is not substantial enough to support these CNV calls as truth sets.”*

-	Fig. 5, which callers achieved relatively better recall and precision should be described in the main text.

*Response: Thank you very much for your comments and suggestions. We have added the description of caller performance in the revised manuscript, as follows:*

*“The accuracy is primarily dependent on the specific caller employed. CNVkit demonstrated relatively higher performance in detecting copy number gain, whereas HATCHet and Control-FREEC exhibited comparatively lower performance. DRAGEN achieved high performance in detect both copy number gain and copy number loss, except for FFPE WGS datasets.”*

-	Fig. S7, C and D, what are the y-axis labels, the same as A and B?

*Response: Yes, a note on Y-axis for figure C and D has been added.*

-	In the Method section, under 'Reproducibility calculation', no justification was provided to exclude certain regions.

*Response: Thank you very much for your comments and suggestions. We have provided explanations for the exclusion of these regions as follows:*

*“The exclusion of the p-arm of chr6, q-arm of chr16, chrX is based on our previous study, where we confirmed that these regions are entirely lost in the genome of HCC1395.The exclusion of gap regions is attributed to the difficulties associated with accurately calling CNVs due to misalignment of NGS short reads.”*

-	In the Method section, under 'Scoring and categorization of CNV calls', the explanation is unclear, and the inclusion of some equations could help provide further clarification.

*Response: Thank you very much for your comments and suggestions. We have revised this section and Figure S4B in the revised manuscript to enhance clarity.*

Reviewer #3: This paper conducted comprehensive comparisons between different copy number variation (CNV) callers, CNV platforms/technologies, and other non-analytical factors to benchmark performances of different pipelines in profiling the CNV patterns in terms of detection accuracy, sensitivity, and reproducibility. The results are very interesting as well as important to help the cancer researchers choose the correct pipelines including analytical tools in their studies. However, because lots of concepts and procedures were proposed for diverse comparisons, some parts of the study were not well organized and conveyed in a neat fashion, making the whole manuscript clumsy and disorienting. Further improvements on the intelligibility of the description of the whole work would be very helpful for the readers with little CNV background knowledge to better understand and appreciate the importance of the paper. Below are several of my other comments/concerns.

*Response: Thank you very much for your insightful comments. We greatly appreciate the acknowledgment of the significance of this study. Following the publication of reference call sets for SNVs and SVs, we have observed a growing interest in CNV call sets for benchmarking purposes. This paper aims to meet this demand by employing a comparable approach to our previous study, establishing a consensus call set, and subsequently validating it using multiple orthogonal methods. Our goal in presenting this CNV reference call set is to offer a valuable resource to the research community, aiding in the advancement and refinement of new tools.*

*Per your suggestion, we have reorganized the structure of the manuscript and improved the descriptions to enhance clarity and understanding.*

(1) The study is well designed as described in the first part of the Results. But the corresponding figure (Fig 1) is somehow confusing and vague. I could not obtain clear information from Fig 1 without reading the text about the study design details.

*Response: Thank you very much for your comments and suggestions. We have updated Figure 1 to make the study design clearer.*

(2) "CNV cluster" seems an important concept in the study, as described in the Method "A CNV cluster is defined as an interval supported by N callsets". However, very fewer results/discussions about CNV cluster were presented.

*Response: Thank you very much for your comments and suggestions. We have replaced the term “cluster” with “interval” in the revised manuscript and illustrated the CNV intervals when defining CNV benchmark sets in Figure S4B.*

(3) In the FFPE datasets, it was good that the authors chose samples with different cell fixing time. Did any results show that the optimal cell fixing time from the perspective of the best calling quality? The fixing time is emphasized in the study design, but its relevance to the calling quality was not adequately described in the results and discussion.

*Response: Thank you for the suggestion. We've examined the calling consistency based on FFPE fixing time, as depicted in Supplementary Figure S11. Our analysis indicates that an increase in FFPE fixing time does not notably influence the accuracy of CNV calls. Moreover, within the FFPE samples, we observed higher accuracy in CNV calls in WGS datasets as opposed to WES datasets. Notably, the* *accuracy is significantly influenced by the specific caller utilized. For instance, CNVkit exhibited excellent performance in detecting copy number gains, while HATCHet and Control-FREEC demonstrated comparatively lower performance levels.*

Figure R10. The F1 score for copy number gain (A) and copy number loss (B) under varying FFPE fixing time.

(4) In Fig 2, an upset plot of genome region of gain/loss called by each CNV caller will be very helpful (similar to Fig 3C)

*Response: Thank you for the suggestion. Considering the volume of up to 126 datasets that require display, the upset plot might not be the optimal method to effectively showcase the consistency of calling results by each caller. We believe that retaining the original plot with stacked bars would present the same message in a more direct and straightforward manner.*

(5) An additional table including 6 CNV callers and key parameters used in the study would be very informative.

*Response: Thank you for the suggestion. A table with key parameters for each caller was added to the supplementary information (Table S4).*

**Second round of review**

**Reviewer 1**

Thank you for addressing the majority of my concerns and making your truth set available for review.
Unfortunately, I strongly disagree with your decision not to reconcile your previously published SV truth set with this CNV truth set. While the overall segments have been orthogonally validated, the start/end positions have not and no attempt has been made to refine them. Segment start/end is extremely important for CN resolution and essential for any high-resolution CN tool/benchmarking.
Justifying this lack of consistency was your results showing that most SVs were small and CNV segments large. Given this, I was quite surprised to find your CNV truth segments are (mostly) not rounded and, to a casual observer, purport to be base-pair accurate.
A quick perusal of your truth bed (I happened to scroll to chr5), and I see that you:
- have a 31bp(!) chr5:12041-12072 segment. That's shorter than the widely used 50bp cut-off for defining SVs. Why do you include tiny CN segments in a truth set that supposedly doesn't have the resolution to be consistent with 1kbp SVs?
- between chr5:18427276 and 18507636, copy number jumps between 4.5 and 5 a total of 13 times. I checked in IGV and I could find no ONT support (https://ftp.ncbi.nlm.nih.gov/ReferenceSamples/seqc/Somatic_Mutation_WG/analysis/SVs/BAMs/tumor.ont.bam) in your data for any of these transitions. A quick google search indicates that UMCCR also thinks these are FP transitions (https://umccr.github.io/umccrise/SEQC-II-50pc__SEQC-II_Tumor_50pc_cancer_report.html). Are you confident that these are genuine copy number transitions and, if not, why include them in your truth set?
- Some segments have suspiciously round start/end positions (chr5:700000-800000) indicating something like 1kbp or 10kbp resolution, but most start/end at seemingly random positions. What defines the CN resolution? What is the smallest CN segment size that you are confident your truth set includes?
As a tool author, I'm going to have to deal with this truth set regardless of where it is published so I'd really like it to be as useful as possible. I'd really like to avoid the scenario where I'm benchmarking tools and the better tool performs worse purely because the truth set is 'wrong'. To make this useful for benchmarking modern CNV tools, I need:
1) Segment start/end confidence intervals
2) A REF/CN=2 track
3) A high-confidence region track (like the NIST HG002 benchmarks)
4) Removal of the CN noise in the gain/loss tracks. If you're not confidence of the actual CN then either add CN CIs or just don't report it. Tools are going to get unfairly penalized for things like not including the CN=4.5 noise segments in chr5:18427276-18507636. Better to not include them in your truth if they're not actually part of your CN segmentation.
5) Make the nominal CN start/end best-guess positions. Ideally you'd match these up with the SV positions but even if you don't, there's less than 700 CN segments so it's only a few days of effort to manually curate them in IGV (@ 5min/segment). If you say a segment starts at chr5:700000 then there's an implicit expectation that the position is approximate. If you say it's chr5:47061388-47069162 then those positions need to be as close as possible to the true CN segment bounds.
6) ASCN (if you have it - currently it looks like you only have total CN + LOH)
7) A VCF version encoding all of the above into a single file would be great since it can encode all of this information.

**Reviewer 2**

I extend my appreciation to the authors for their meticulous attention to my initial concerns and suggestions. Through their comprehensive responses and the meticulous revisions made to the manuscript, it is evident that they have effectively addressed each of my queries, significantly enhancing the quality and clarity of their work.

The authors have notably strengthened the manuscript by providing detailed explanations for the selection of the six CNV callers, distinguishing between somatic and germline CNV detection, elucidating cutoff value criteria, quantifying concordance levels, clarifying methodological approaches, and refining figure legends. These enhancements have not only improved the manuscript's comprehensiveness but also provided valuable insight into the rationale behind their methodologies.

Additionally, I commend the authors for their commitment to transparency and reproducibility by making all supplementary data publicly available. This step further solidifies the integrity and impact of their research and underscores their dedication to advancing the field.

Overall, the authors have conducted an exhaustive and high-quality benchmarking analysis of somatic CNV detection tools, providing a valuable resource for the cancer genomics community. Their responsiveness to my comments and the subsequent revisions have fully addressed my concerns.

**Reviewer 3**

All my questions have been addressed. The authors have invested significant efforts in enhancing the manuscript with additional information including more figures. Thank you.

**Authors Response**

**Point-by-point responses to the reviewers’ comments:**

**Reviewer #1:**

Thank you for addressing the majority of my concerns and making your truth set available for review.

**Concern #1:**

Unfortunately, I strongly disagree with your decision not to reconcile your previously published SV truth set with this CNV truth set. While the overall segments have been orthogonally validated, the start/end positions have not and no attempt has been made to refine them. Segment start/end is extremely important for CN resolution and essential for any high-resolution CN tool/benchmarking.

***Response:*** *In the previous round of review, we clarified that the reference SV call set may capture events occurring in a small population of HCC1395 cells detectable by any tools, whereas the CNV reference call set defined in this study represents the chromosome copy number status in the major cell population. Additionally, in our paper (**Talsania et al., Genome Biology 23(1), 255, 2022), we presented sequencing results from both short-read and long-read technologies, as they were intended to detect SV across a spectrum of fragment sizes. However, the CNV call set was solely based on short-read technology using a consensus approach. Consequently, the SV call set would include a greater number but smaller segment sizes of deletions (DEL) and duplications (DUP). It is therefore crucial to maintain their separation to prevent potential misuse in benchmarking or validating new tools. In fact, a recent publication by Wang et al. (Nat Biotechnol., March 2024) utilized our SV reference call set to validate their newly developed tool, SVision-pro, for de novo and somatic structural variant discovery.*

*To alert reader for the use of our CNV reference call set, we have added the following statement in the Discussion section.*

*“It worth to note that we previously defined structural variant (SV) call set encompassing various types, such as DEL (deletion), DUP (duplication), INS (insertion), INV (inversion), TRA (translocation), and BND (Complex Break Point Events) [12]. The SV detection callers primarily sought supporting reads for these events, focusing on higher resolutions that are more sensitive to sub-populations of cells. Previous studies have indicated the highly heterogeneous nature of the cancer cell line HCC1395, characterized by large subclones. Consequently, SV events occurring in smaller cell populations were incorporated into the final SV reference call set, resulting in a larger number of events defined in SV (1788) compared to CNV (688) (Supplementary file 1). Notably, the average sizes of DEL (deletion) and DUP (duplication), equivalent to LOSS and GAIN respectively, were considerably smaller [12]. In contrast, CNV callers primarily targeted the LOSS or GAIN of chromosome regions within overall cell populations, factoring in genome ploidy. Therefore, CNV calling exhibited lower sensitivity towards events represented by a small fraction of cells or a limited number of supporting reads. Consequently, we assert that the SV and CNV call sets should remain separate entities.”*

**Concern #2:**

Justifying this lack of consistency was your results showing that most SVs were small and CNV segments large. Given this, I was quite surprised to find your CNV truth segments are (mostly) not rounded and, to a casual observer, purport to be base-pair accurate.

A quick perusal of your truth bed (I happened to scroll to chr5), and I see that you:

-	have a 31bp(!) chr5:12041-12072 segment. That's shorter than the widely used 50bp cut-off for defining SVs. Why do you include tiny CN segments in a truth set that supposedly doesn't have the resolution to be consistent with 1kbp SVs?

-	between chr5:18427276 and 18507636, copy number jumps between 4.5 and 5 a total of 13 times.

I checked in IGV and I could find no ONT support (https://ftp.ncbi.nlm.nih.gov/ReferenceSamples/seqc/Somatic_Mutation_WG/analysis/SVs/BAMs/tumor.ont.bam) in your data for any of these transitions. A quick google search indicates that UMCCR also thinks these are FP transitions (https://umccr.github.io/umccrise/SEQC-II-50pc__SEQC-II_Tumor_50pc_cancer_report.html). Are you confident that these are genuine copy number transitions and, if not, why include them in your truth set?

-	Some segments have suspiciously round start/end positions (chr5:700000-800000) indicating something like 1kbp or 10kbp resolution, but most start/end at seemingly random positions. What defines the CN resolution? What is the smallest CN segment size that you are confident your truth set includes?

***Response:*** *Thank you very much for pointing that out. The example referenced was within a large gain segment spanning 16,405,027 base pairs, which exhibited high amplification. The copy number presented in the table was derived from the median value of copy number calls made by six different callers. However, due to the inconsistency of copy number calls by these six callers within this region, the gain segment was further subdivided based on copy number. We acknowledge that this approach may not have been the optimal method for defining copy number in our original reference CNV call set. Per your request, we have addressed this in the second version of the manuscript. Nevertheless, we have included the following statement in the Discussion section.*

“*We also wish to point out that even though we provided copy number calls in the reference call set (Supplementary file 2 and 3), our confidence was only limited to qualification of CNV calls, i.e, GAIN, LOSS, and LOH. The copy number calls which were derived from the average of called values by six callers should not be used as “gold-standard” for benchmarking study*.”

*Since the approach we employed to define copy number was somewhat arbitrary, we do not consider 4.5 and 5 to be significantly different. Therefore, we rounded up the final copy number for gain segments to a whole number and merged continuous segments with the same copy number calls. This resulted in a large number of fragmented segments being consolidated into larger regions. As a consequence, only three segments are now defined between chr5:18427276 and 18507636 (Table 1).*

| Chromosome | start | end | copy number |
| --- | --- | --- | --- |
| chr5 | 16416909 | 17530548 | 4 |
| chr5 | 17580548 | 18427276 | 4 |
| chr5 | 18427276 | 18600000 | 5 |

Table 1, after consolidation, only one segment with CNV # calls defined between chr5:18427276 and 18507636

*Highly amplified regions were also observed in chr14 (105900000 – 106300000), chr2 (88850000 – 89250000), possibly explained as extrachromosomal DNA due to chromothripsis, since 64-79 chromosomes were observed in the HCC1395 cell line by cell karyotyping (Fang et al, Nature Biotech., 2021, Extended Data Fig. 5).*

*As indicated in our previous response, we did not utilize ONT for either the initial CNV definition or the confirmation of the final reference call set. Due to insufficient coverage and sequence quality of ONT reads, we solely employed ONT reads to aid genome assembly (Xiao et al., Genome Biology 23(1), 237, 2022).*

*We observed that CNV results displayed by UMCCR were derived from Manta, adjusted using PURPLE. Manta was primarily designed for detecting germline structural variants (Chen et al., Bioinformatics, 32(8), 2016). The limitations of using Manta and PURPLE for somatic CNV calling have been previously discussed in the peer review process.*

*The start and end positions of segments were predominantly inherited from the original calling results by each caller. Since we utilized a window size of 50,000kb during the analysis by CNV callers, rounded start/end positions in the reference CNV call set were expected.*

*Upon your suggestion, we implemented a cutoff of 50bp for CNV size. Consequently, a total of 5 segments, including one gain segment, were removed from our reference CNV call set (Table 2).*

| Chromosome | start | end | CNV call | size |
| --- | --- | --- | --- | --- |
| chr1 | 39014556 | 39014558 | gain | 2 |
| chr1 | 40933358 | 40933408 | loh | 50 |
| chr2 | 10174295 | 10174338 | loh | 43 |
| chr6 | 1.02E+08 | 1.02E+08 | loh | 43 |
| chr10 | 4834090 | 4834133 | loh | 43 |

Table 2, five segments with size less than 50 bp were removed from the reference call set

**Concern #3:**

As a tool author, I'm going to have to deal with this truth set regardless of where it is published so I'd really like it to be as useful as possible. I'd really like to avoid the scenario where I'm benchmarking tools and the better tool performs worse purely because the truth set is 'wrong'. To make this useful for benchmarking modern CNV tools, I need:

1)	Segment start/end confidence intervals

2)	A REF/CN=2 track

3)	A high-confidence region track (like the NIST HG002 benchmarks)

***Response:*** *We have included 4 supplementary files containing clear definitions of segment start/end positions for the reference CNV call set, encompassing GAIN, LOSS, and LOH events, along with genes located in the affected regions.*

*We omitted the definition of neutral segments (CN=2) since not all orthogonal technologies report copy-neutral segments in their results. Without rigorous test, we do not feel comfortable to include CN=2 in the reference CNV call set.*

*Users can utilize the defined segment regions as a high-confidence CNV track for their benchmarking studies.*

**Concern #4:**

4)	Removal of the CN noise in the gain/loss tracks. If you're not confidence of the actual CN then either add CN CIs or just don't report it. Tools are going to get unfairly penalized for things like not including the CN=4.5 noise segments in chr5:18427276-18507636. Better to not include them in your truth if they're not actually part of your CN segmentation.

***Response:*** *Please see our response to concern #2.*

**Concern #5:**

5)	Make the nominal CN start/end best-guess positions. Ideally you’d match these up with the SV positions but even if you don’t, there’s less than 700 CN segments so it’s only a few days of effort to manually curate them in IGV (@ 5min/segment). If you say a segment starts at chr5:700000 then there’s an implicit expectation that the position is approximate. If you say it’s chr5:47061388-47069162 then those positions need to be as close as possible to the true CN segment bounds.

***Response:*** *As previously indicated in our response to concern #2, the segment start and end positions were primarily inherited from the original calling results by each caller, considering a window size of 50,000kb during the analysis. Consequently, rounded start/end positions in the reference CNV call set were expected. However, we also employed a consensus approach to define high-confidence CNV regions and merged/extended CNV regions using clusters of segments, which allowed for the generation of more precise positions.*

*As you suggested, we performed manual review of the CNV call set on IGV and determined that the following segments should be removed due to lack of sufficient evidence (Table 3). As a result, a total of 210 kb regions were removed from the final reference CNV call set, which corresponded for less than 0.01% of the total CNV regions called for the cell line. Therefore, we don’t believe this change will affect our previous conclusions.*

| Chromosome | Start | End | Size | Call | Supported | Notes |
| --- | --- | --- | --- | --- | --- | --- |
| chr4 | 68550000 | 68600000 | 50000 | gain | No | This appears to be copy-neutral or LOSS |
| chr5 | 11882 | 12041 | 159 | gain | No | Tiny segment near telomere with high coverage, flanking region is GAIN, probably correct but low confidence call |
| chr8 | 39450000 | 39528723 | 78723 | gain | No | Consistent with copy-neutral |
| chr10 | 39230136 | 39238955 | 8819 | gain | No | Consistent with copy-neutral |
| chr10 | 39239118 | 39254773 | 15655 | gain | No | Consistent with copy-neutral |
| chr10 | 39338450 | 39341685 | 3235 | gain | No | Consistent with copy-neutral |
| chr17 | 21976531 | 21983452 | 6921 | loh | No | Few BAF sites and largely around BAF ~ 50%, alignment bad in this region, low confidence |
| chr17 | 22042061 | 22089188 | 47127 | loh | No | Few BAF sites and largely around BAF ~ 50%, high-identity segdup, difficult to call |

Table 3, segments without sufficient support via manual review on IGV

*While we did conduct manual examination of segment boundaries using IGV, this review was limited to identifying any obvious discrepancies in read coverage, b-allele frequency, and CNV breakpoints. Unless significant discrepancies were observed, which was not the case, we did not manually adjust CNV breakpoints.*

**Concern #6:**

6)	ASCN (if you have it – currently it looks like you only have total CN + LOH)

***Response:*** *Thank you for the suggestion. However, we do not believe the available data would empower such analysis. It is indeed our plan for future improvement to incorporate data from the latest technologies to enable more advanced analyses like ASCN.*

**Concern #7:**

7)	A VCF version encoding all of the above into a single file would be great since it can encode all of this information

***Response:*** *Thank you for the suggestion. A VCF version of supplementary file was included.*

**Third round of review**

**Reviewer 1**

Thank you for the updates addressing my concerns. While it is disappointing not to have a REF/CN=2 track or confidence intervals around the CN segment start/end, it is still a useful truth set for CNV calling. There are a few final corrections that would be useful before publication.

1) LOSS/LOH consistency
chr21 36949289 LOSS:chr21:36949289-36951263 N <LOSS> . . END=36951263;CN=1;CNVLEN=1974 GT ./.
chr21 36951263 LOSSLOH:chr21:36951263-37899646 N <LOSS>,<LOH> . . END=37899646;CN=1;CNVLEN=948383 GT ./.
chr21 37899646 LOSS:chr21:37899646-38900000 N <LOSS> . . END=38900000;CN=1;CNVLEN=1000354 GT ./.

Not sure how that middle segment is LOSS and LOH and the other two are just LOSS. They're all CN=1. Unless there's subclonality, LOSS/CN=1 implies LOH. Either make the notation consistent, or explicitly state that LOSS without LOH implies that there is indeed no LOH and there's subclonality at play (and what the min AF of that would be).

2) start/end confidence interval

Add an extra sentence somewhere in the paper explicitly stating how accurate the segment start/end bounds are. Ideally you'd just add confidence intervals but you don't appear to have the data for that.

3) Update the VCF to be specifications-compliant. Specifically:
-- Add the the IMPRECISE flag (defined to be "variants with an unspecified confidence interval") or add CIPOS/CIEND values
-- Use SVLEN instead of CNVLEN
-- Use <DEL> <DUP> with SVCLAIM=D or Use <CNV> subtypes (e.g. <CNV:GAIN>) so specifications-complaint VCF libraries will treat these records as CNV records instead of custom symbolic alleles with no specifications-defined meaning.
-- Add SVTYPE=CNV (if using VCFv4.4 or earlier)
-- Add CICN if you have the data for it
-- Add the missing Type=Integer from the ##INFO=<ID=CN, header line
-- LOH isn't standardised (seems like an omission in the VCF specs themselves) so your encoding is acceptable. If you had explicit CN=2 REF segments I would recommend defining LOH has an INFO flag but doesn't really work for your LOH-only records such as LOH:chr1:2800000-4067592.

**Authors Response**

**Point-by-point responses to the reviewers’ comments:**

*Response:*

*Concern #1:*

*Followed suggestion and take out LOH annotation of LOSS (CN=1). As a results, three segments are combined as a bigger segment at chr21: 36949289-38900000*

*Concern #2:*

*Added following statement in the discussion section: " In addition, break points of CNV segment were the best estimate and thus imprecise. "*

*Concern #3:*

*Followed reviewer's suggestion on additional flags.*
